# Supplementary material for: Protective Immune Signatures Associated with Latent TB Infection in PLHIV—Insights from an Integrative Prospective Immune Monitoring Study
Source: Cells. 2025 Oct 17;14(20):1622. doi: 10.3390/cells14201622 (PMC12562479; doi:10.3390/cells14201622)

Supplementary file:

Table S1: Detailed table for clinical, demographic, Immunological and virological characteristics of study population

Sr.no	Sample ID	LTBI status	HIV status	Age (years)	Gender	CD4 (cells/µL)	CD8 (cells/µL)	CD4/CD8	Viral load (copies/mL)	Other clinical history
1	IU-0001	Positive	Positive	39	M	228	638	0.36	127797	None
2	IU-0002	Positive	Positive	52	M	NA	NA	NA	364446	None
3	IU-0003	Positive	Positive	NA	M	126	356	0.35	103230	None
4	IU-0009	Positive	Positive	45	F	360	671	0.537	17227	None
5	IU-0010	Positive	Positive	38	M	156	1341	0.12	143686	Active TB
6	IU-0011	Positive	Positive	53	M	836	1237	0.68	NA	None
7	IU-0012	Positive	Positive	30	F	10	572	0.02	376315	None
8	IU-0014	Positive	Positive	18	F	348	1518	0.23	150666	Extrapulmonary Active TB stomach
9	IU-0015	Negative	Positive	50	M	NA	NA	NA	156209	None
10	IU-0016	Positive	Positive	41	M	201	2257	0.09	328950	Active TB
11	IU-0017	Positive	Positive	36	M	607	390	1.56	5621	History of TB in 2015
12	IU-0018	Positive	Positive	41	M	381	1087	0.35	63403	None
13	IU-0019	Positive	Positive	40	M	343	878	0.39	217655	None
14	IU-0020	Negative	Positive	36	F	62	3280	0.02	1062802	History of TB
15	IU-0021	Negative	Positive	48	M	78	682	0.11	685254	None
16	IU-0022	Negative	Positive	30	M	449	3847	0.12	23404	None
17	IU-0023	Negative	Positive	50	F	63	522	0.12	678124	Active TB
18	IU-0024	Positive	Positive	44	F	146	883	0.17	163566	Active TB
19	IU-0025	Negative	Positive	NA	M	6	1073	0.01	10440	None
20	IU-0026	Negative	Positive	34	M	168	1457	0.12	97122	Active TB
21	IU-0027	Negative	Positive	50-60	M	79	464	0.17	237231	Suspected Active TB
22	IU-0028	Positive	Positive	52	M	285	2263	0.13	2878	Diabetes
23	IU-0029	Negative	Positive	45	M	318	992	0.32	111105	None
24	IU-0030	Positive	Positive	37	M	369	843	0.44	28633	None
25	IU-0031	Positive	Positive	42	M	NA	NA	NA	38242	History of TB 5 years back
26	IU-0032	Positive	Positive	38	M	454	948	0.48	200347	Acidity, Pain in body
27	IU-0033	Negative	Positive	32	F	158	582	0.27	47965	Hyperthyroidism
28	IU-0034	Positive	Positive	50	F	123	1620	0.08	372086	Pulmonary TB
29	IU-0035	Negative	Positive	37	M	629	880	0.7	329073	Perianal abscess
30	IU-0036	Positive	Positive	59	M	486	808	0.6	63530	Diabetes & high BP
31	IU-0037	Positive	Positive	43	F	409	1047	0.39	562529	Ovarian Cyst

Sr.no	Sample ID	LTBI status	HIV status	Age (years)	Gender	CD4 (cells/µL)	CD8 (cells/µL)	CD4/CD8	Viral load (copies/mL)	Other clinical history
32	IU-0038	Negative	Positive	56	F	124	990	0.12	21042	None
33	IU-0039	Negative	Positive	40	F	38	685	0.05	256062	High blood pressure, fever, difficulty in breathing
34	IU-0040	Negative	Positive	49	F	201	1473	0.13	46811	Gastritis
35	IU-0041	Positive	Positive	53	M	277	1486	0.18	329635	Diabetes
36	IU-0044	Negative	Positive	24	M	615	1551	0.39	26651	Depression
37	IU-0045	Positive	Positive	52	F	468	2333	0.2	75238	None
38	IU-0046	Negative	Positive	31	M	199	620	0.32	93670	None
39	IU-0047	Positive	Positive	27	M	617	961	0.64	391914	VDRL +ve, Skin Boils, Ulcers in mouth
40	IU-0048	Positive	Positive	40	M	49	551	0.08	11110633	Active TB
41	IU-0049	Negative	Positive	37	M	605	861	0.7	159237	None
42	IU-0050	Positive	Positive	31	M	72	500	0.14	621950	None
43	IU-0051	Negative	Positive	52	F	166	1097	0.15	301915	Breast cancer, High BP, Diabetes
44	IU-0052	Negative	Positive	41	M	52	809	0.06	700909	Active TB
45	IU-0053	Negative	Positive	51	M	70	1304	0.05	509272	Hemorrhoids and Active TB
46	IU-0054	Negative	Positive	30	M	599	1090	0.54	21035	History of COVID-19 two months prior blood collection
47	IU-0055	Negative	Positive	39	M	499	903	0.55	88054	None
48	IU-0056	Negative	Positive	52	M	214	951	0.22	574791	None
49	IU-0057	Negative	Positive	50	M	127	326	0.38	2331565	Active TB
50	IU-0058	Positive	Positive	38	F	364	1091	0.33	90982	Diabetes
51	IU-0059	Positive	Positive	37	M	281	1237	0.22	861227	Active TB
52	IU-0060	Negative	Positive	33	M	350	1533	0.22	84502	Hemorrhoids
53	IU-0061	Positive	Positive	40	F	360	1406	0.26	130558	None
54	IU-0062	Negative	Positive	40	M	28	530	0.05	160677	Active TB
55	IU-0068	Positive	Positive	50	F	90	1314	0.06	257156	Active TB
56	IU-0069	Negative	Positive	52	F	205	1039	0.19	49715	High blood pressure
57	IU-0070	Positive	Positive	34	M	455	825	0.55	682	Active TB
58	IU-0071	Positive	Positive	43	F	481	707	0.7	28031	None
59	IU-0072	Negative	Positive	23	F	709	736	0.96	69886	None

Sr.no	Sample ID	LTBI status	HIV status	Age (years)	Gender	CD4 (cells/µL)	CD8 (cells/µL)	CD4/CD8	Viral load (copies/mL)	Other clinical history
60	IU-0073	Negative	Positive	41	M	50	578	0.09	725869	None
61	IU-0075	Positive	Positive	42	M	NA	NA	NA	29279	None
62	IU-0077	Negative	Positive	28	M	835	1694	0.49	11727	None
63	IU-0078	Negative	Positive	35	M	132	791	0.17	20699	None
64	IU-0080	Negative	Positive	30	M	761	2280	0.33	22571	None
65	IU-0081	Negative	Positive	47	M	47	817	0.06	2138	None
66	IU-0082	Negative	Positive	30	M	625	1343	0.47	64655	None
67	IU-0083	Negative	Positive	47	F	474	455	1.04	1099	None
68	IU-0084	Positive	Positive	36	M	5	410	0.01	NA	Extrapulmona-ry TB
69	IU-0086	Negative	Positive	41	F	435	1551	0.28	44975	None
70	IU-0092	Negative	Positive	49	F	135	530	0.26	5920711	None
71	IU-0093	Negative	Positive	36	F	461	844	0.55	34835	Operated for Gall bladder stones
72	IU-0094	Negative	Positive	59	M	136	536	0.25	1520377	Active TB
73	IU-0097	Negative	Positive	33	M	325	618	0.53	658	None
74	IU-0100	Negative	Positive	24	M	528	5164	0.1	217676	Hemorrhoids
75	IU-0101	Positive	Positive	30	M	849	1632	0.52	7775	None
76	IU-0102	Positive	Positive	29	M	325	601	0.54	227876	None
77	IU-0104	Negative	Positive	25	M	68	885	0.08	647390	Mild hepatomegaly, multiple small non-necrotic mesenteric and para aortic lymphnodes
78	IU-0105	Positive	Positive	41	M	213	464	0.46	654	Lymphadenopathy
79	IU-0107	Positive	Positive	29	M	200	587	0.34	231836	None
80	IU-0108	Positive	Positive	42	F	87	665	0.13	74674.87	Diabetes, cervical Lymphadenopathy, Active TB
81	IU-0109	Negative	Positive	50	F	330	1049	0.31	24.91162	Hearing loss
82	IU-0110	Positive*	Positive	40	M	20	227	0.09	126.882	None
83	IU-0111	Positive	Positive	33	F	223	594	0.38	183.288	Weakness and fever
84	IU-0112	Negative	Positive	18	F	427	1706	0.25	22.3314	None
85	IU-0114	Negative	Positive	58	M	147	1743	0.08	158.34	None
86	IU-0115	Negative	Positive	39	F	748	594	1.26	1.68462	Stomach ache
87	IU-0116	Negative	Positive	42	F	448	3887	0.12	1184.82	None
88	IU-0121	Positive	Positive	26	M	653	2869	0.228	996.24	None

Sr.no	Sample ID	LTBI status	HIV status	Age (years)	Gender	CD4 (cells/µL)	CD8 (cells/µL)	CD4/CD8	Viral load (copies/mL)	Other clinical history
89	IU-0122	Positive	Positive	45	M	1127	1293	0.871	33.4824	Malaria
90	IU-0123	Negative	Positive	53	M	332	4107	0.081	1945.44	None
91	IU-0124	Indeterminant	Positive	42	M	61	360	0.169	1865.64	Active TB
92	IU-0125	Negative	Positive	19	M	404	1150	0.351	1219.26	Genital boils
93	IU-0126	Positive	Positive	48	M	88	2170	0.04	868.98	Varicose vein, venous ulcer in right leg
94	IU-0127	Positive	Positive	54	M	577	1141	0.506	0.135996	None
95	IU-0128	Positive	Positive	47	F	1021	784	1.303	13.6584	High blood pressure & Diabetes
96	IU-0129	Negative	Positive	50	M	351	319	1.103	48.3	Water retention in lungs
97	IU-0131	Negative	Positive	43	M	84	809	0.1	2515.38	None
98	IU-0132	Positive	Positive	35	M	220	564	0.39	109.074	None
99	IU-0133	Negative	Positive	25	M	146	677	0.22	227.85	None
100	IU-0134	Negative	Positive	34	M	38	458	0.08	137.592	Active TB, Mild splenomegaly and Hepatitis B +ve
101	IU-0136	Negative	Positive	52	F	299	894	0.33	85.092	Cataract
102	IU-0138	Positive	Positive	35	M	230	517	0.45	66.696	None
103	IU-0140	Negative	Positive	30	F	658	1452	0.45	2387.7	None
104	IU-0145	Negative	Positive	29	M	155	1502	0.1	1516.2	Active TB
105	IU-0146	Negative	Positive	31	M	10	254	0.04	7896	None
106	IU-0147	Negative	Positive	27	M	98	712	0.14	679190	None
107	IU-0148	Negative	Positive	26	M	584	1001	0.58	1207.08	None
108	IU-0149	Negative	Positive	26	M	43	1142	0.04	152158	None
109	IU-0150	Negative	Positive	49	F	216	302	0.72	16992	None
110	IU-0151	Negative	Positive	36	M	100	1138	0.09	1871972	None
111	IU-0152	Negative	Positive	37	F	404	607	0.67	6613	None
112	IU-0153	Negative	Positive	43	M	184	888	0.21	221996	None

NA: Not available

Figure S1: Gating strategy for T cells, B cells, NK cells and monocytes counts

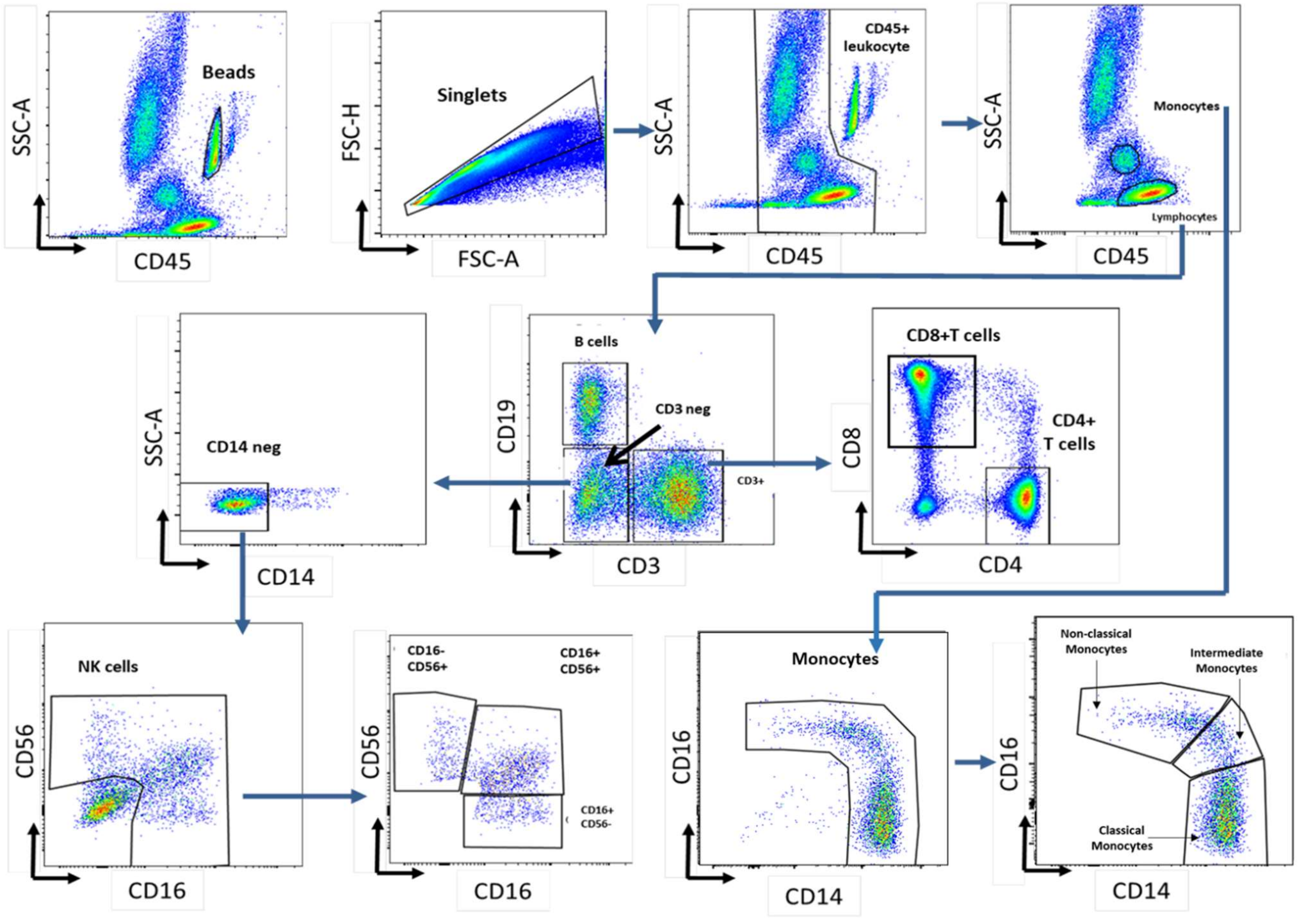

Representative flow cytometric gating strategy for absolute counts of T cells, B cells, NK cells, monocytes and their subsets: First, the bead population was gated from all events. Next we gate on singlets on the basis of FSC-A and FSC-H. Further total leukocytes followed by lymphocytes and monocytes were gated on the basis of CD45 expression. On the lymphocyte population CD3+ T cells are gated on the basis of expression of CD3 and B cells are identified as CD19+ population, apart from these 2 populations we also gate for CD3 negative population. CD8+ and CD4+T cell were identified by the expression of CD4 and CD8 markers. From CD3 negative population we further gate for CD14 negative cells followed by different populations of NK cells on the basis of expression of CD16 and CD56. Lastly from the monocytes population we further identify different types of monocytes on the basis of expression of CD14 and CD16.

Figure S2: Immunological and virological characteristics of study population: Absolute counts of (A) CD4+ T cells and (B) CD8+ T cells. (C) CD4/CD8 ratios (D) viral load. Correlation between viral loads with (E) absolute counts of CD4+ T cells (F) CD4/CD8 ratios. (G) Mean relative expressions of markers for absolute count panel generated using Cluster Explorer Plugin for unsupervised analysis. Comparisons between groups were calculated by Kruskal-Wallis one-way ANOVA non-parametric test, (*p < 0.05; **p < 0.01; ***p < 0.001; ****p < 0.0001). p and r values for associations were determined by Spearman's correlation test and p < 0.05 was considered significant.

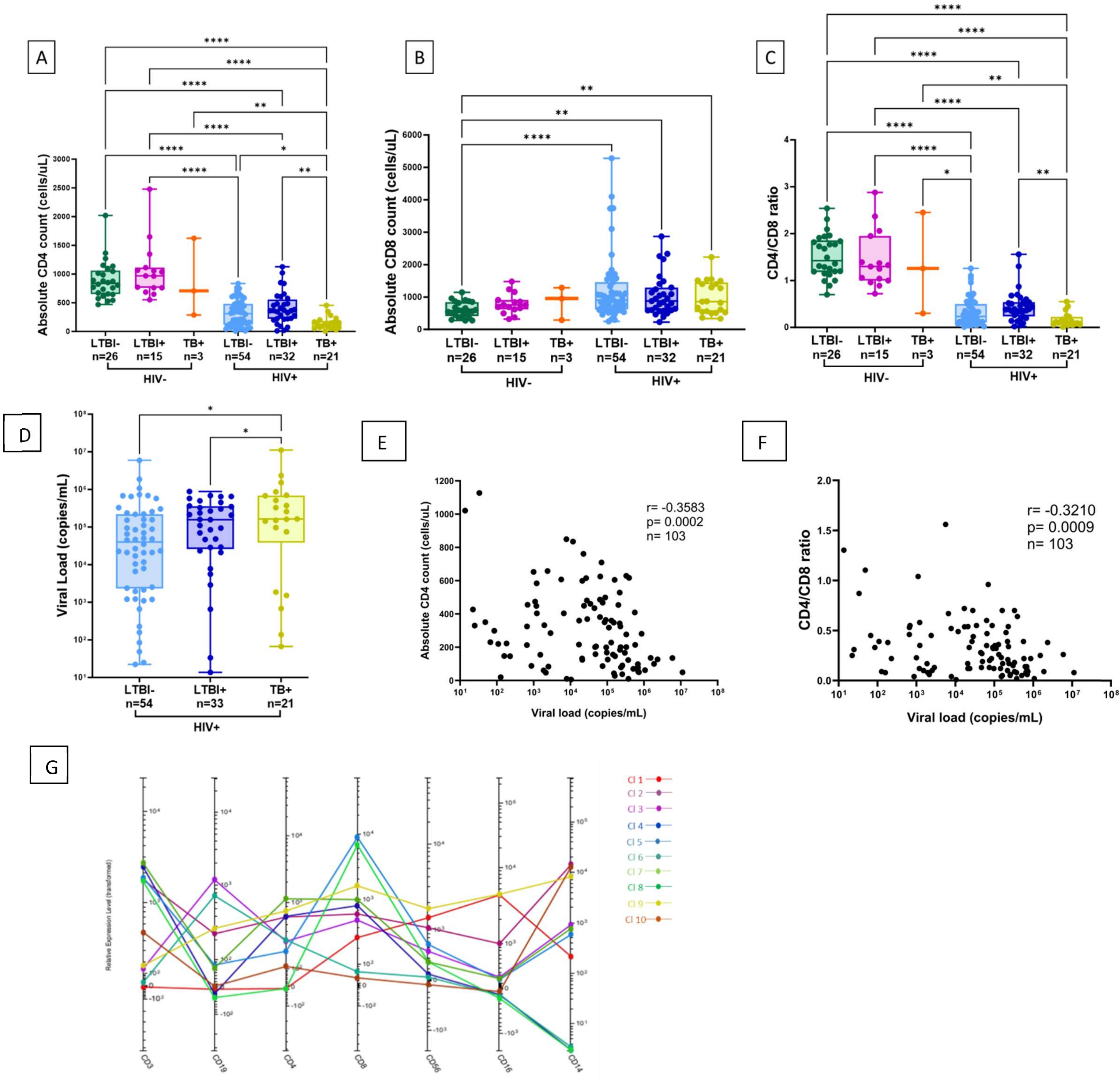

Figure S3A: Gating strategy for activation and PD-1 expression of T cells and subsets

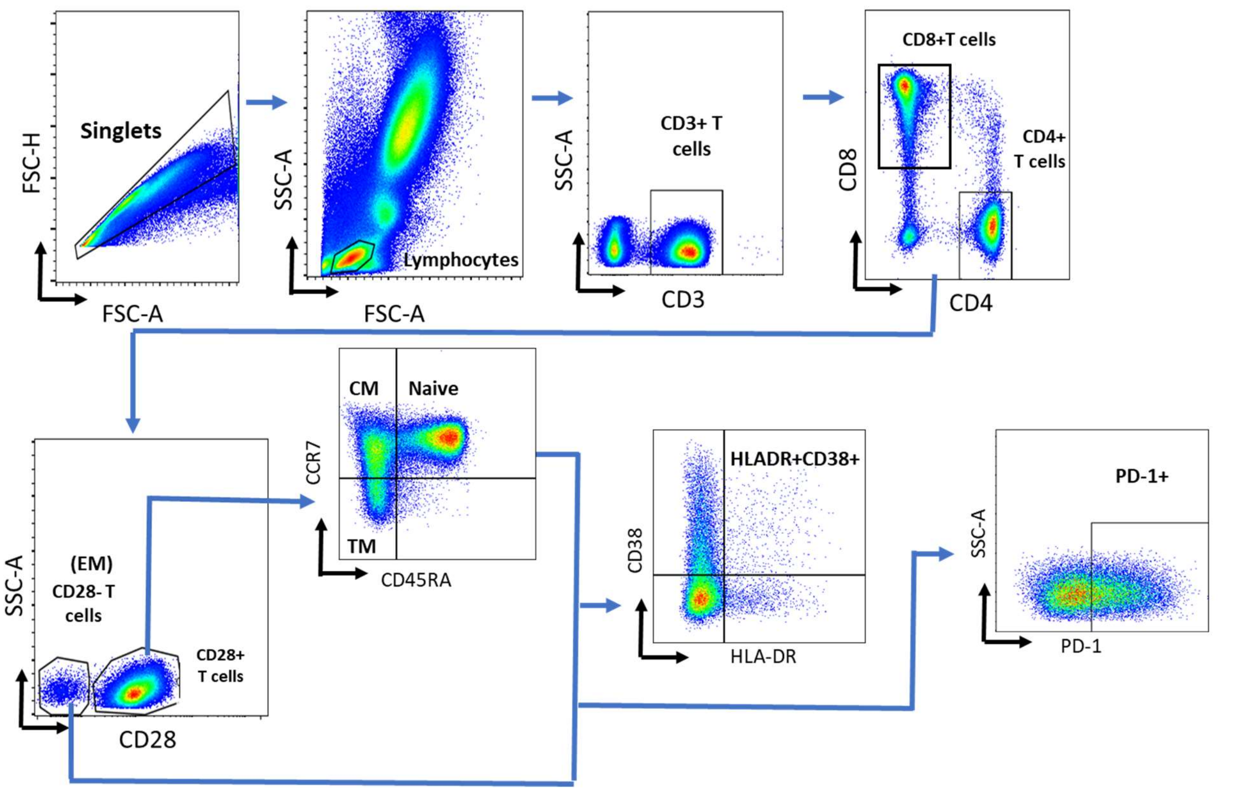

Representative flow cytometric gating strategy for activation and PD-1 expression in T cells and its subsets: First, singlets are gated on the basis FSC-A and FSC-H. Further, lymphocytes are gated on the basis of FSC-A and SSC-A. Following lymphocytes, T cells are gated on the basis of expression of CD3 and subsequently, CD8 and CD4. For both CD4+ and CD8+ T cells, based on expression of CD28, CD45RA and CCR7 naïve, memory and effector T cell subsets were gated. From each naïve/memory subset activated/modulated T cells were identified on the basis of co-expression of HLA-DR, CD38 and that of PD-1 separately.

Figure S3B: Mean relative expressions of markers for T cell subset activation and PD-1 expression generated using Cluster Explorer Plugin for unsupervised analysis

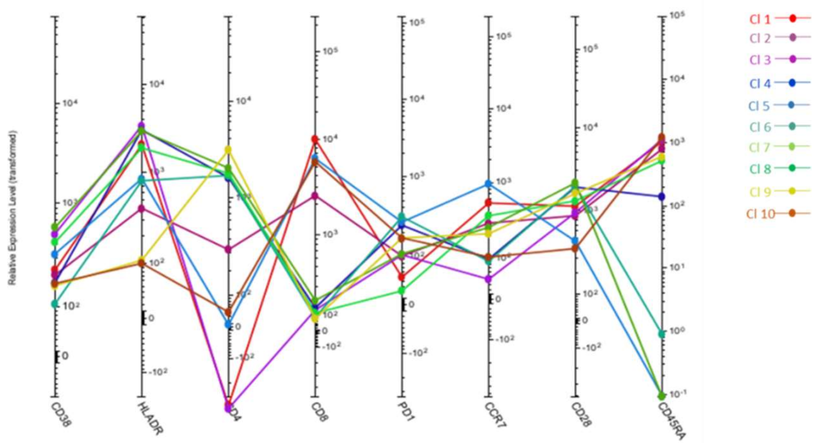

Figure S4A: Gating strategy for regulatory T cells and subsets

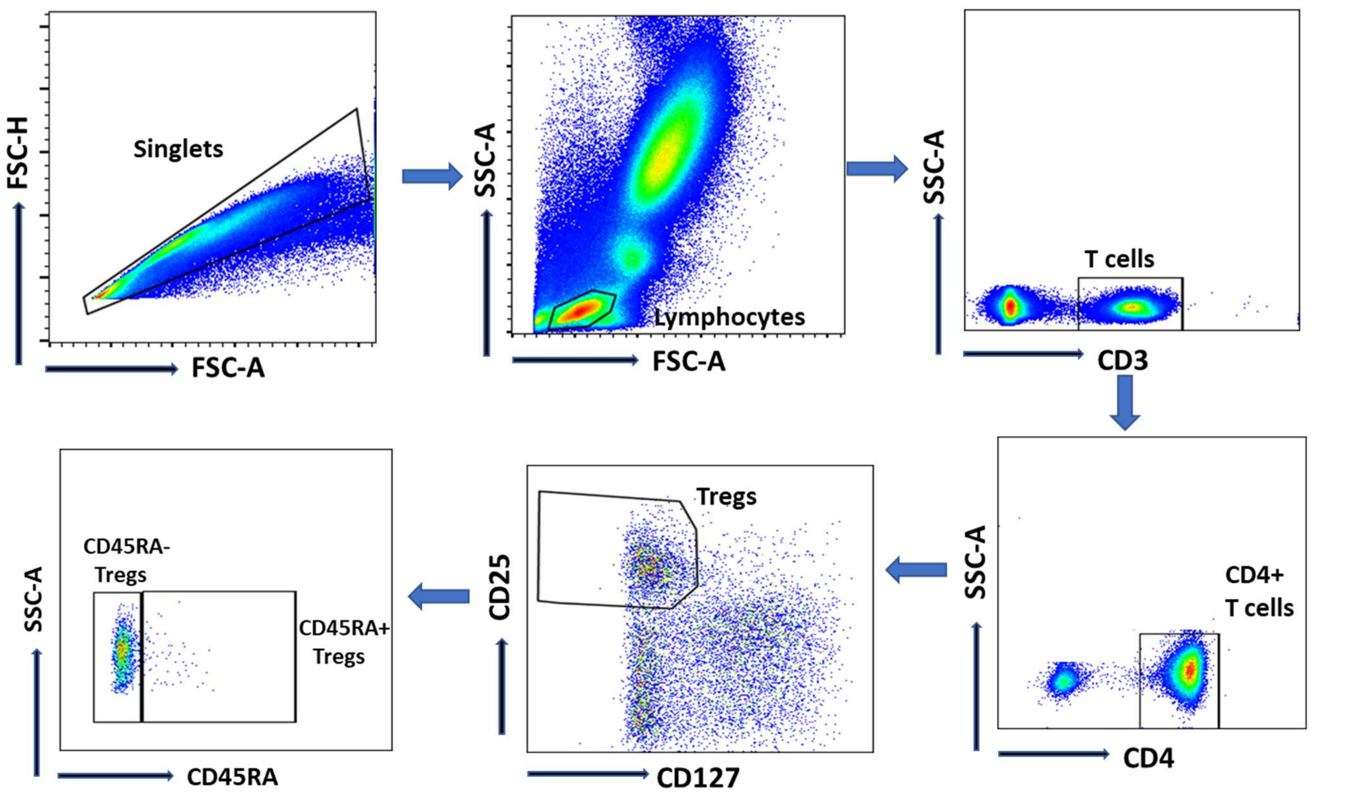

Representative flow cytometric gating strategy for T regulatory subsets: First, singlets were gated on the basis FSC-A and FSC-H. Further, lymphocytes were gated on the basis of FSC-A and SSC-A. Within lymphocytes, T cells were gated on the basis of expression of CD3 and subsequently CD4. Next, based on expression of CD25 (IL-2Rα) and CD127 (IL-7Rα) Tregs were identified and further gated as naïve and memory Tregs based on the expression of CD45-RA.

Figure S4B: Mean relative expressions of markers for Tregs and subsets generated using ClusterExplorerPlugin for unsupervised analysis

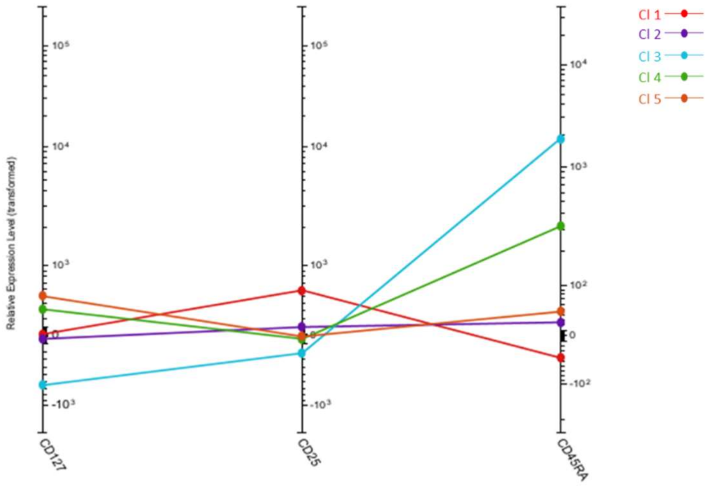

Figure S5: Correlations between Tregs and subsets and disease progression markers: Correlation between CD4 absolute counts with absolute counts of (A) total Treg (B) Naïve Treg (C) Memory Treg and frequency of (D) Total Treg (E) Naïve Treg and (F) Memory Treg. Correlation of Treg frequency with (G) CD4/CD8 ratio (H) Viral load. Correlation of frequency of naive Tregs with (I) CD4/CD8 ratio (J) Viral load. Correlation of frequency of memory Tregs with (K) CD4/CD8 ratio and (L) Viral load. p and r values for associations were determined by Spearman's correlation test, with linear regression shown as a line and $p < 0.05$ was considered significant. Red dots represent HIV+LTBI+ group while black dots represent HIV+LTBI- group.

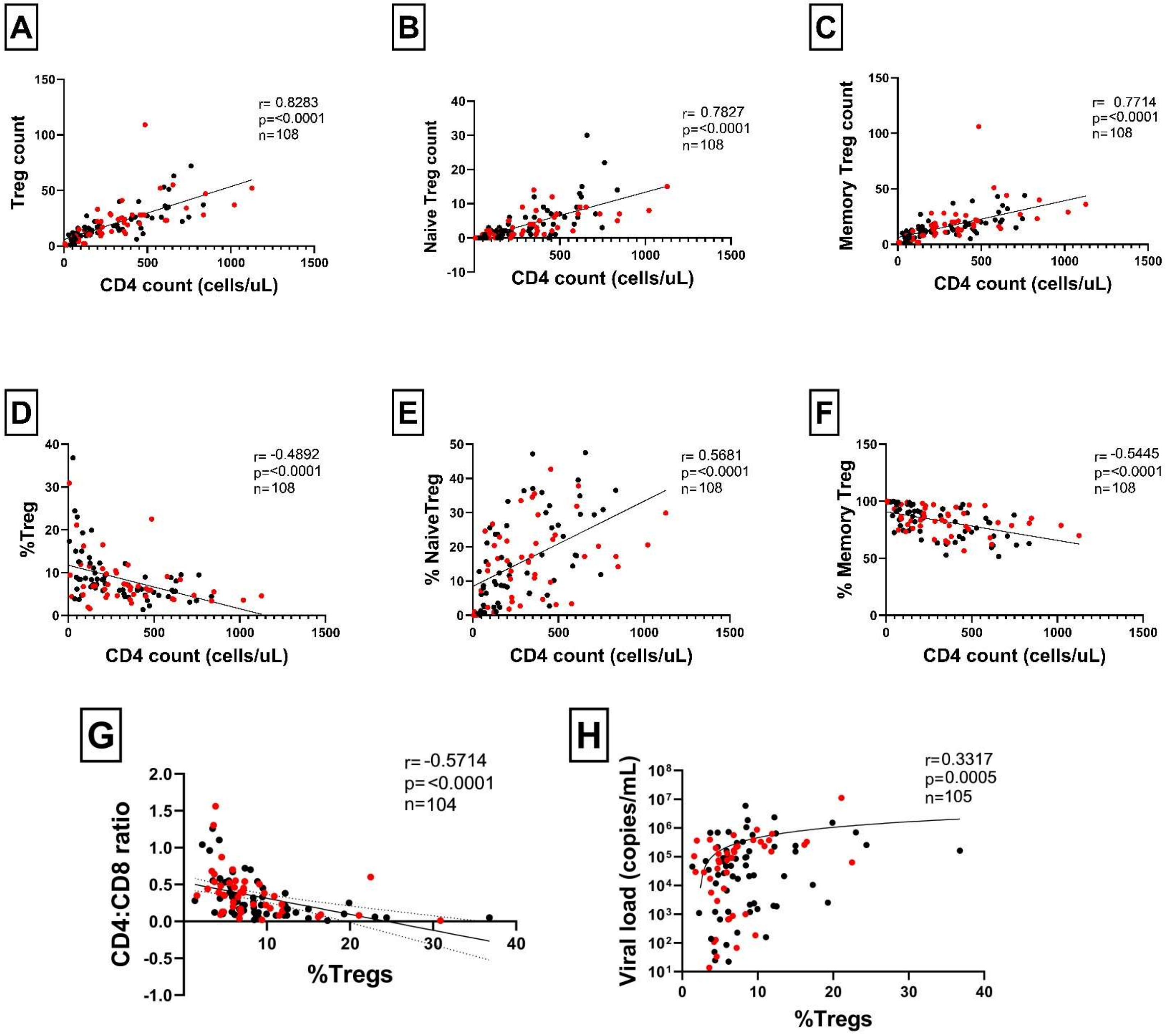

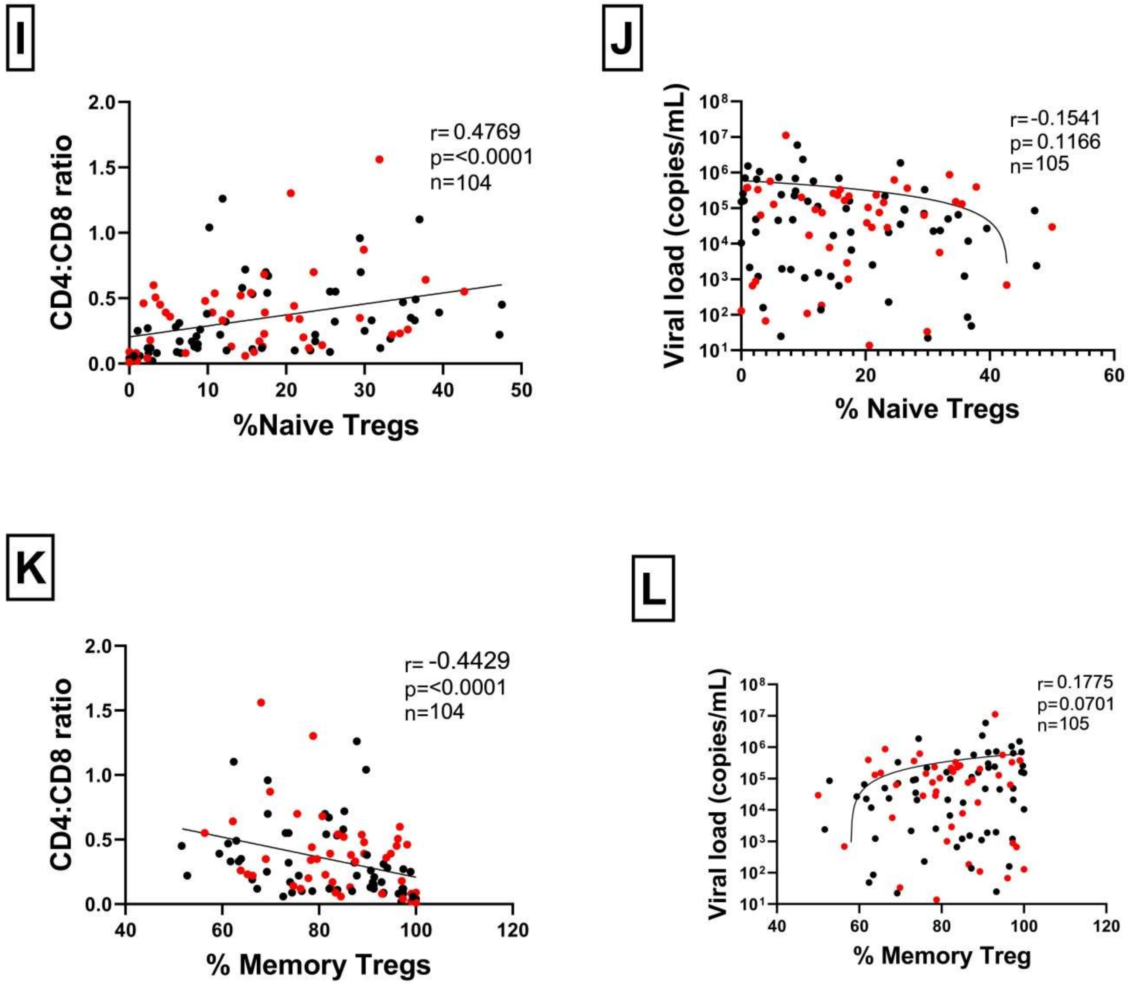

Figure S6: sCD14, sCD163, CRP, D-dimer and cytokine levels in blood plasma: Levels of plasma (A) sCD14 (B) sCD163 (C) CRP in ng/mL and levels of (D) D-dimer (E) IP-10 ~~IL-10~~ (F) IL-12p70 (G) IFN-γ in pg/mL across different HIV sero-negative and HIV-1 positive groups. Comparisons between groups were calculated by Kruskal-Wallis one-way ANOVA non-parametric test, (*p < 0.05; **p < 0.01; ***p < 0.001; ****p < 0.0001)

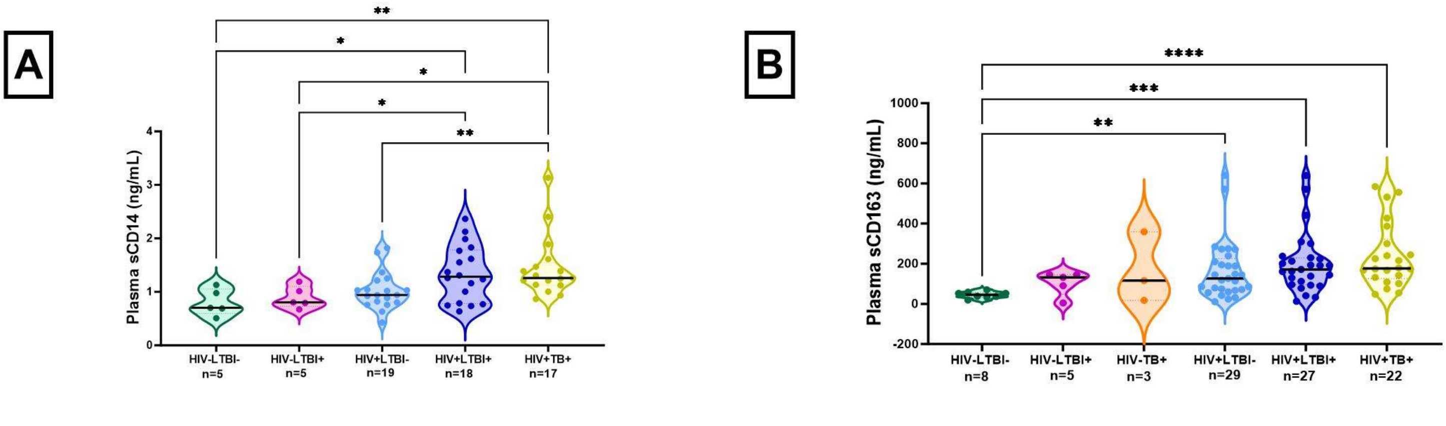

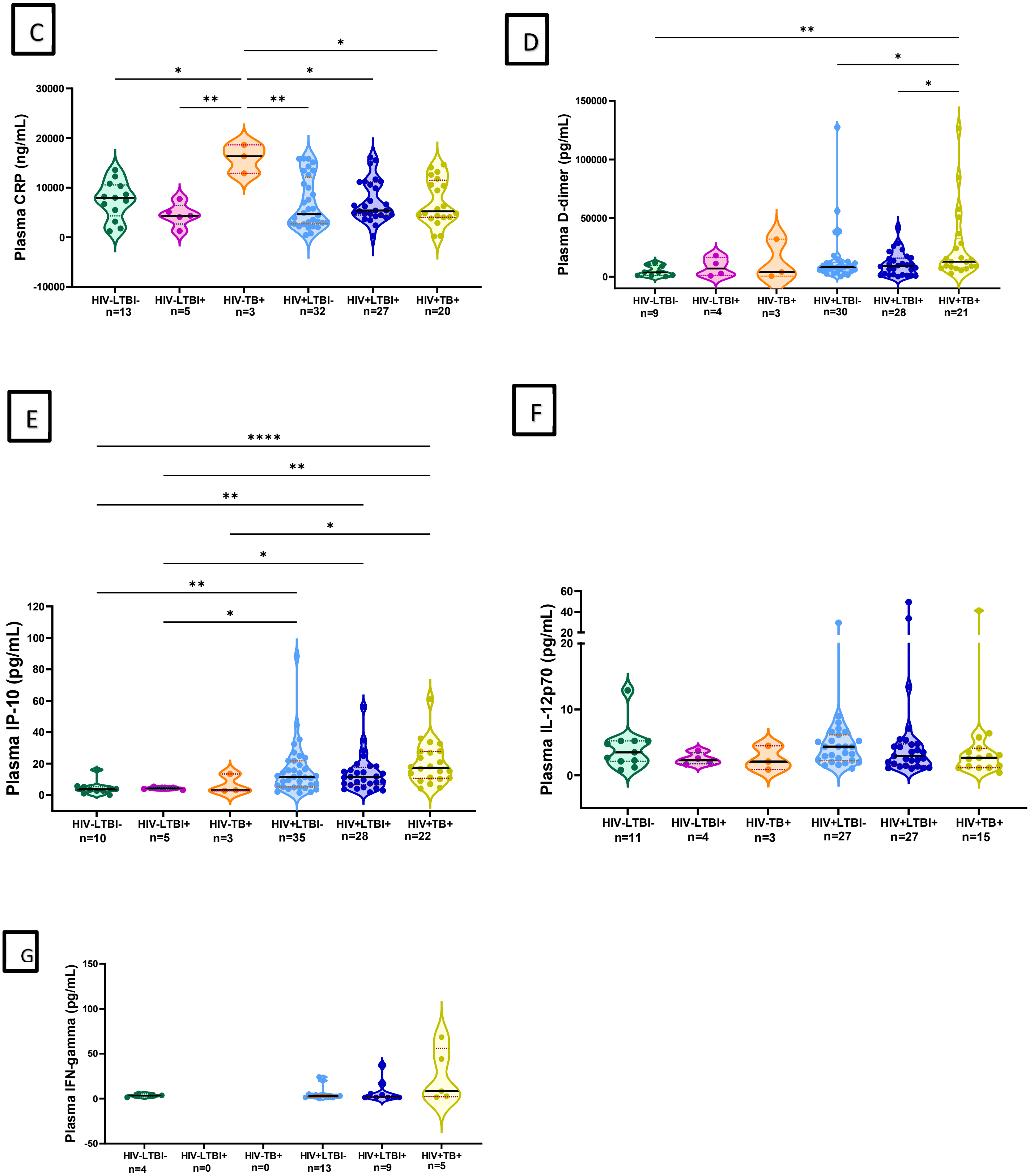
C
Plasma CRP (ng/mL)
30000
20000
10000
0
-10000
HIV-LTBI- n=13
HIV-LTBI+ n=5
HIV-TB+ n=3
HIV+LTBI- n=32
HIV+LTBI+ n=27
HIV+TB+ n=20
D
Plasma D-dimer (pg/mL)
150000
100000
50000
0
HIV-LTBI- n=9
HIV-LTBI+ n=4
HIV-TB+ n=3
HIV+LTBI- n=30
HIV+LTBI+ n=28
HIV+TB+ n=21
E
Plasma IP-10 (pg/mL)
120
100
80
60
40
20
0
HIV-LTBI- n=10
HIV-LTBI+ n=5
HIV-TB+ n=3
HIV+LTBI- n=35
HIV+LTBI+ n=28
HIV+TB+ n=22
F
Plasma IL-12p70 (pg/mL)
60
40
20
20
10
0
HIV-LTBI- n=11
HIV-LTBI+ n=4
HIV-TB+ n=3
HIV+LTBI- n=27
HIV+LTBI+ n=27
HIV+TB+ n=15
G
Plasma IFN-gamma (pg/mL)
150
100
50
0
-50
HIV-LTBI- n=4
HIV-LTBI+ n=0
HIV-TB+ n=0
HIV+LTBI- n=13
HIV+LTBI+ n=9
HIV+TB+ n=5

Figure S7: Hierarchical clustering analysis: Unsupervised clustering analysis for various systemic immune markers (A) Absolute CD4+ T cell count (B) CD4:CD8 ratio (C) Activation of CD4+ and CD8+ T cells (D) PD-1 expression on CD8+ T cells and (E) T regulatory cells.

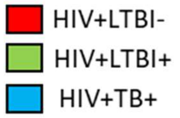

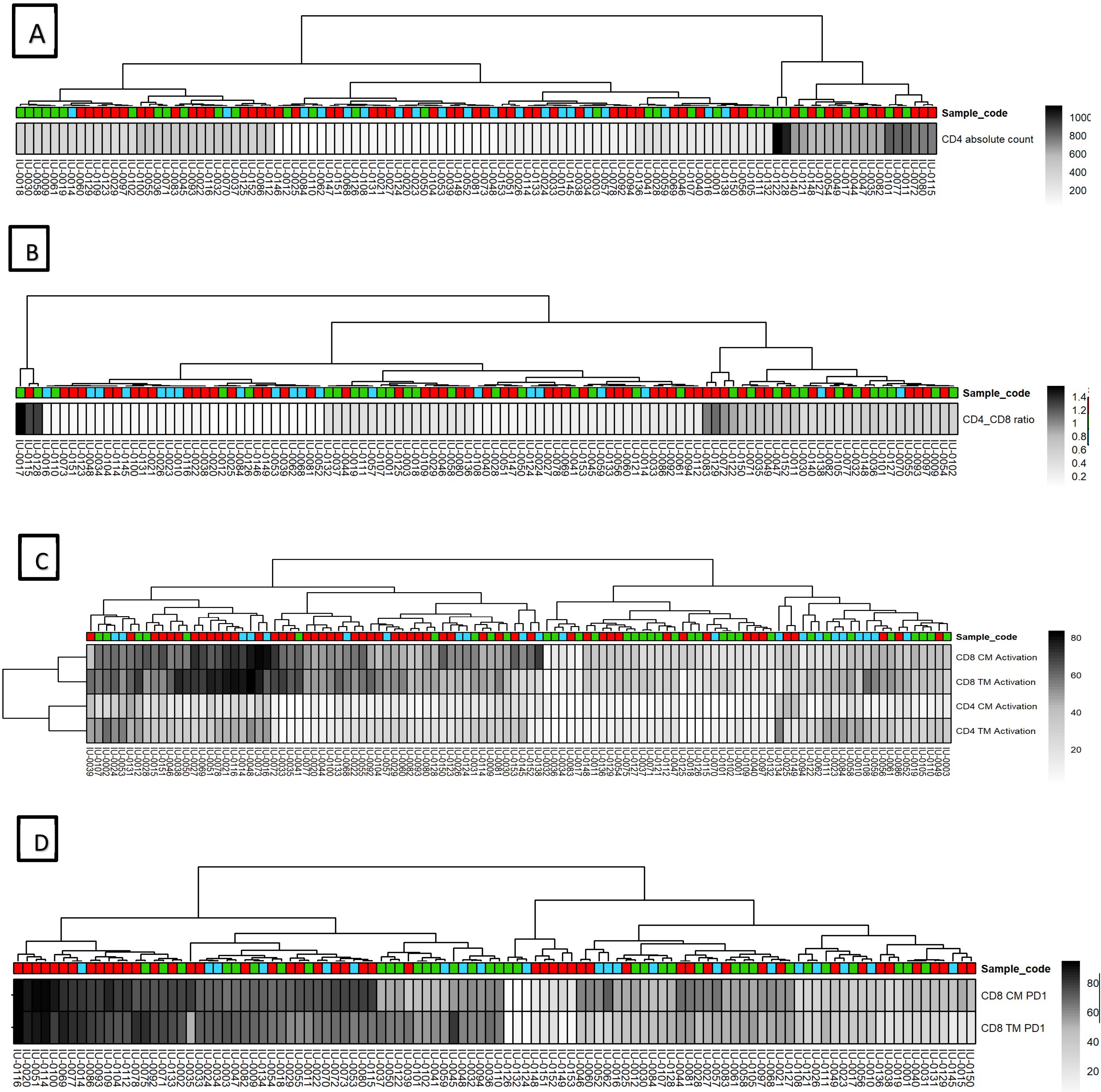

E

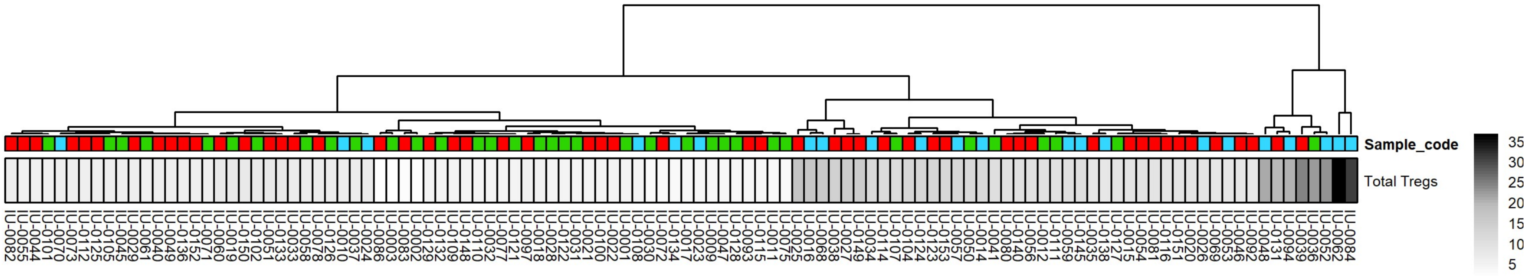

Table S2: Distribution of outlier samples in HLTBI+ group from hierarchical clustering analysis

	Percentile								
**Sample ID**	**CD4 absolute counts**	**CD4:CD8 ratio**	**CD4+ CM activation**	**CD4+ TM activation**	**CD8+ CM activation**	**CD8+ TM activation**	**CD8+ CM PD1+**	**CD8+ TM PD-1+**	**Treg**	**Viral Load copies/mL**
**IU-0002**	NA	NA	75-100th	75-100th	75-100th	75-100th	75-100th	75-100th	0-25th	75-100th
**IU-0009**	25-50th	75-100th	75-100th	50-75th	50-75th	50-75th	75-100th	75-100th	0-25th	25-50th
**IU-0012**	25-50th	0-25th	75-100th	75-100th	75-100th	75-100th	50-75th	25-50th	75-100th	75-100th
**IU-0030**	50-75th	50-75th	75-100th	75-100th	75-100th	50-75th	0-25th	0-25th	0-25th	25-50th

Footnote: NA- Not available

Figure S8: Status of disease progression markers following ART initiation in HLTBI+ individuals: Unmatched data for absolute counts of (A) CD4+ T cells and (B) CD8+ T cells. (C) CD4/CD8 ratio and (D) viral load. Matched data for absolute counts of (E) CD4+ T cells and (F) CD8+ T cells. (G) CD4/CD8 ratios and (H) viral load. Comparison between groups for unmatched data was calculated by Kruskal-Wallis one-way ANOVA non-parametric test, (*p < 0.05). Comparison between TP0 and TP1 of matched data was calculated by Wilcoxon matched-pairs signed rank test (*p < 0.05, **p < 0.01, ***p < 0.001).

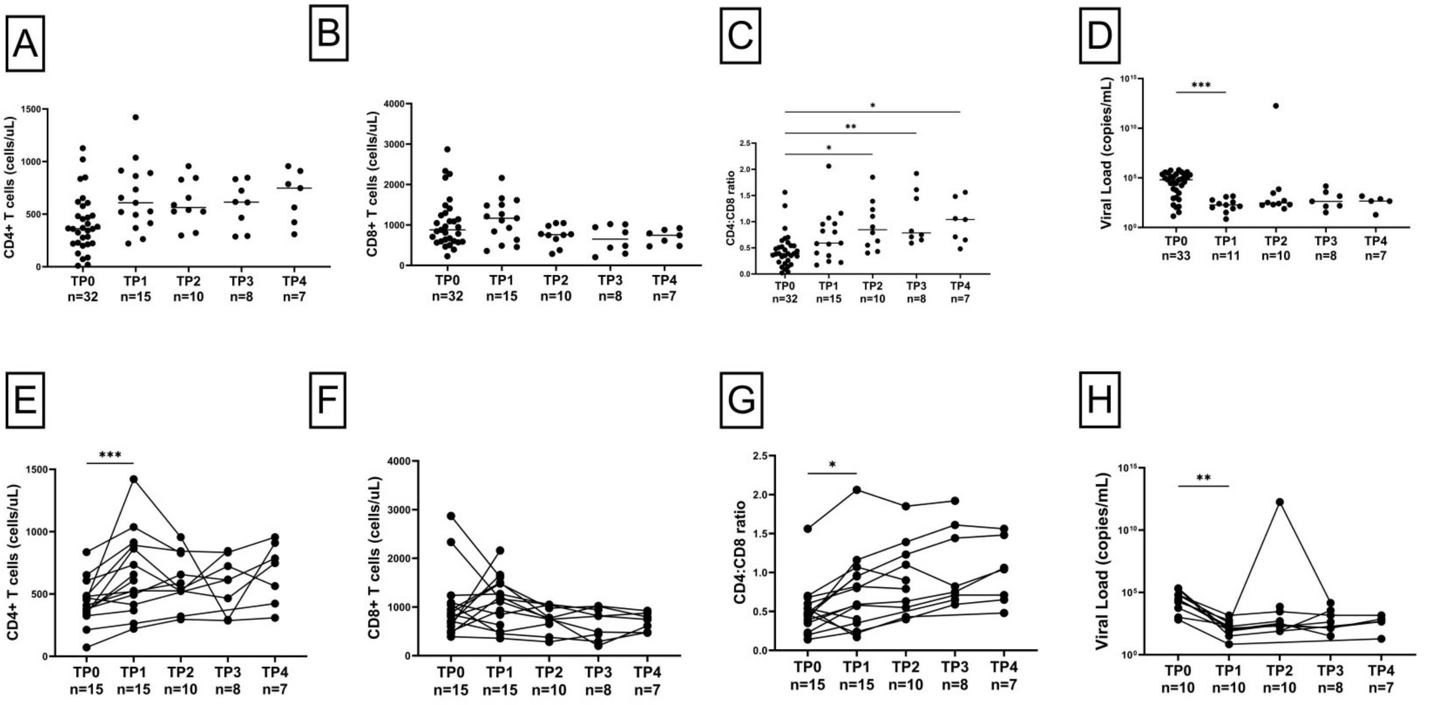

Figure S9: Levels of activation following ART initiation in HLTBI+ individuals: Responder rate for (A) CD4+ T cell activation levels and (B) CD8+ T cell activation levels. Matched data for frequency of activated (HLADR+ CD38+) CD4+ T cell subsets of (C) Naive (D) Central memory (E) Transitional memory and (F) Effector memory and in CD8+ T cell subsets of (G) Naive (H) Central memory (I) Transitional memory and (J) Effector memory at TP1, TP2 and TP3. Unmatched data for frequency of activated (HLADR+ CD38+) CD4+ T cell subsets of (K) Naive (L) Central memory (M) Transitional memory and (N) Effector memory and in CD8+ T cell subsets of (O) Naive (P) Central memory (Q) Transitional memory and (R) Effector memory at TP1, TP2 and TP3. Area shaded in beige color represent of range of frequency of HIV sero-negative levels and grey dotted line represent median. Comparison for matched data between groups was calculated by Friedman one-way ANOVA non-parametric test, and for unmatched data was calculated by Kruskal-Wallis one-way ANOVA non-parametric test (*p < 0.05; **p < 0.01; ***p < 0.001; ****p < 0.0001)

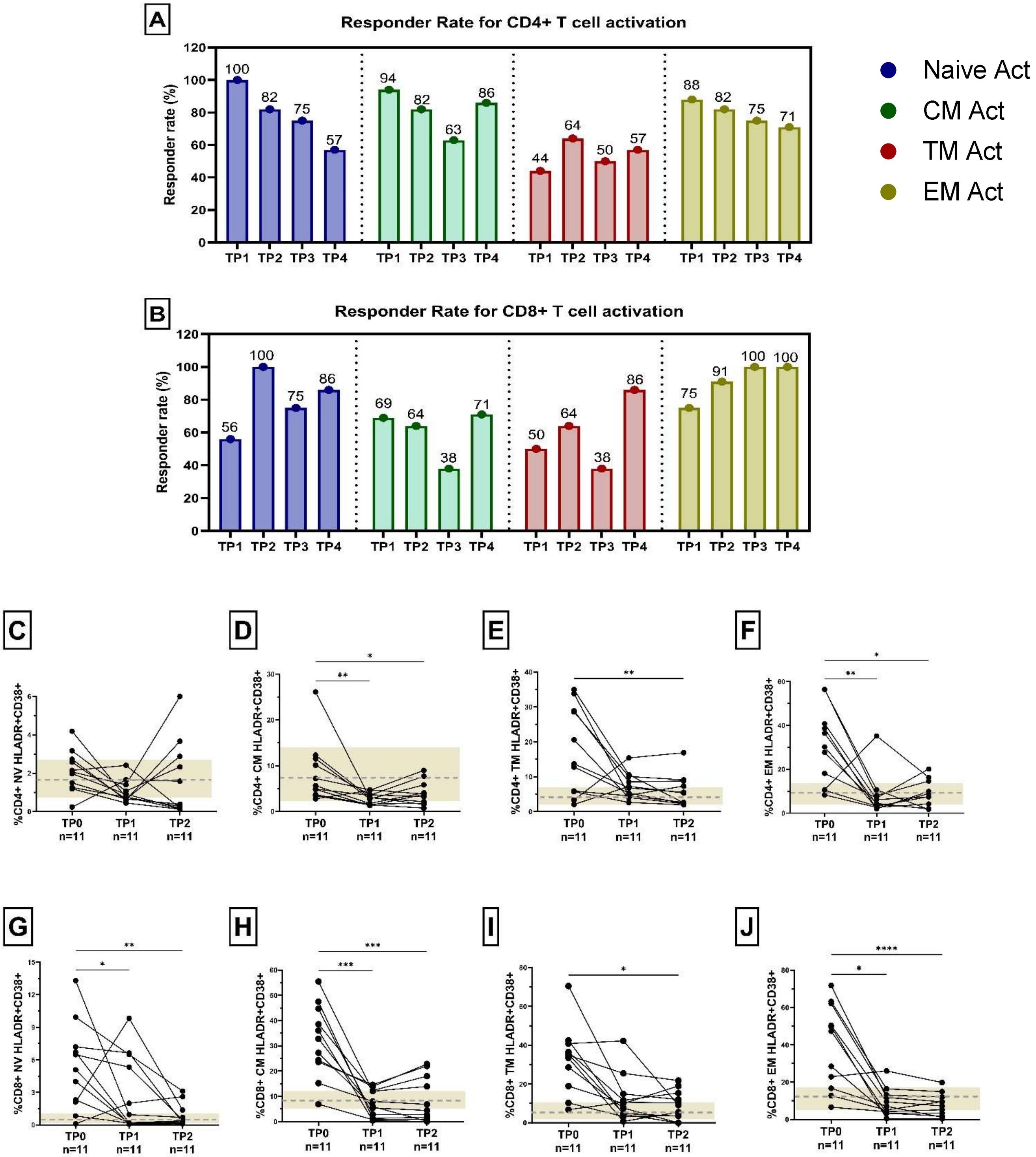

A
Responder Rate for CD4+ T cell activation
Responder rate (%)
100
82
75
57
94
82
63
86
44
64
50
57
88
82
75
71
TP1 TP2 TP3 TP4
Naive Act
CM Act
TM Act
EM Act
B
Responder Rate for CD8+ T cell activation
56
100
75
86
69
64
38
71
50
64
38
86
75
91
100
100
C
%CD4+ NV HLADR+CD38+
D
%CD4+ CM HLADR+CD38+
E
%CD4+ TM HLADR+CD38+
F
%CD4+ EM HLADR+CD38+
G
%CD8+ NV HLADR+CD38+
H
%CD8+ CM HLADR+CD38+
I
%CD8+ TM HLADR+CD38+
J
%CD8+ EM HLADR+CD38+
TP0 n=11
TP1 n=11
TP2 n=11

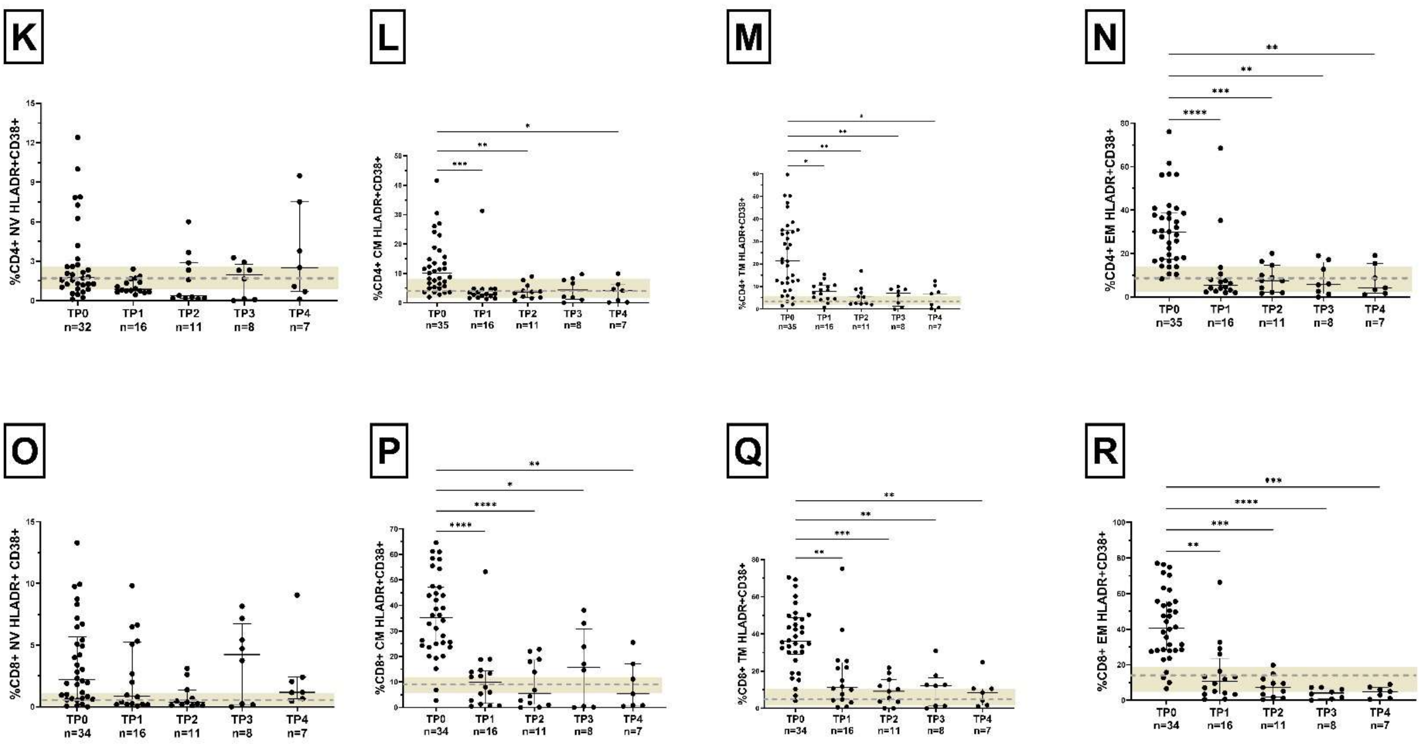

K
%CD4+ NV HLADR+CD38+
TP0 n=32
TP1 n=16
TP2 n=11
TP3 n=8
TP4 n=7
L
%CD4+ CM HLADR+CD38+
TP0 n=35
TP1 n=16
TP2 n=11
TP3 n=8
TP4 n=7
M
%CD4+ TM HLADR+CD38+
TP0 n=35
TP1 n=16
TP2 n=11
TP3 n=8
TP4 n=7
N
%CD4+ EM HLADR+CD38+
TP0 n=35
TP1 n=16
TP2 n=11
TP3 n=8
TP4 n=7
O
%CD8+ NV HLADR+ CD38+
TP0 n=34
TP1 n=16
TP2 n=11
TP3 n=8
TP4 n=7
P
%CD8+ CM HLADR+CD38+
TP0 n=34
TP1 n=16
TP2 n=11
TP3 n=8
TP4 n=7
Q
%CD8+ TM HLADR+CD38+
TP0 n=34
TP1 n=16
TP2 n=11
TP3 n=8
TP4 n=7
R
%CD8+ EM HLADR+CD38+
TP0 n=34
TP1 n=16
TP2 n=11
TP3 n=8
TP4 n=7

Figure S10: Levels of PD-1 expression following ART initiation in HLTBI+ individuals: Responder rate for (A) PD-1 expression in CD4+ T cell and (B) PD-1 expression in CD8+ T cell activation levels. Matched data for frequency of PD-1+ CD4+ T cell subsets of (C) Naive (D) Central memory (E) Transitional memory and (F) Effector memory and CD8+ T cell subsets of (G) Naive (H) Central memory (I) Transitional memory and (J) Effector memory at TP1, TP2 and TP3. Area shaded in cream color represent of range of frequency of HIV sero-negative levels and grey dotted line represent median. Comparison between groups was calculated by Friedman one-way ANOVA non-parametric test, (*p < 0.05; **p < 0.01; ***p < 0.001; ****p < 0.0001)

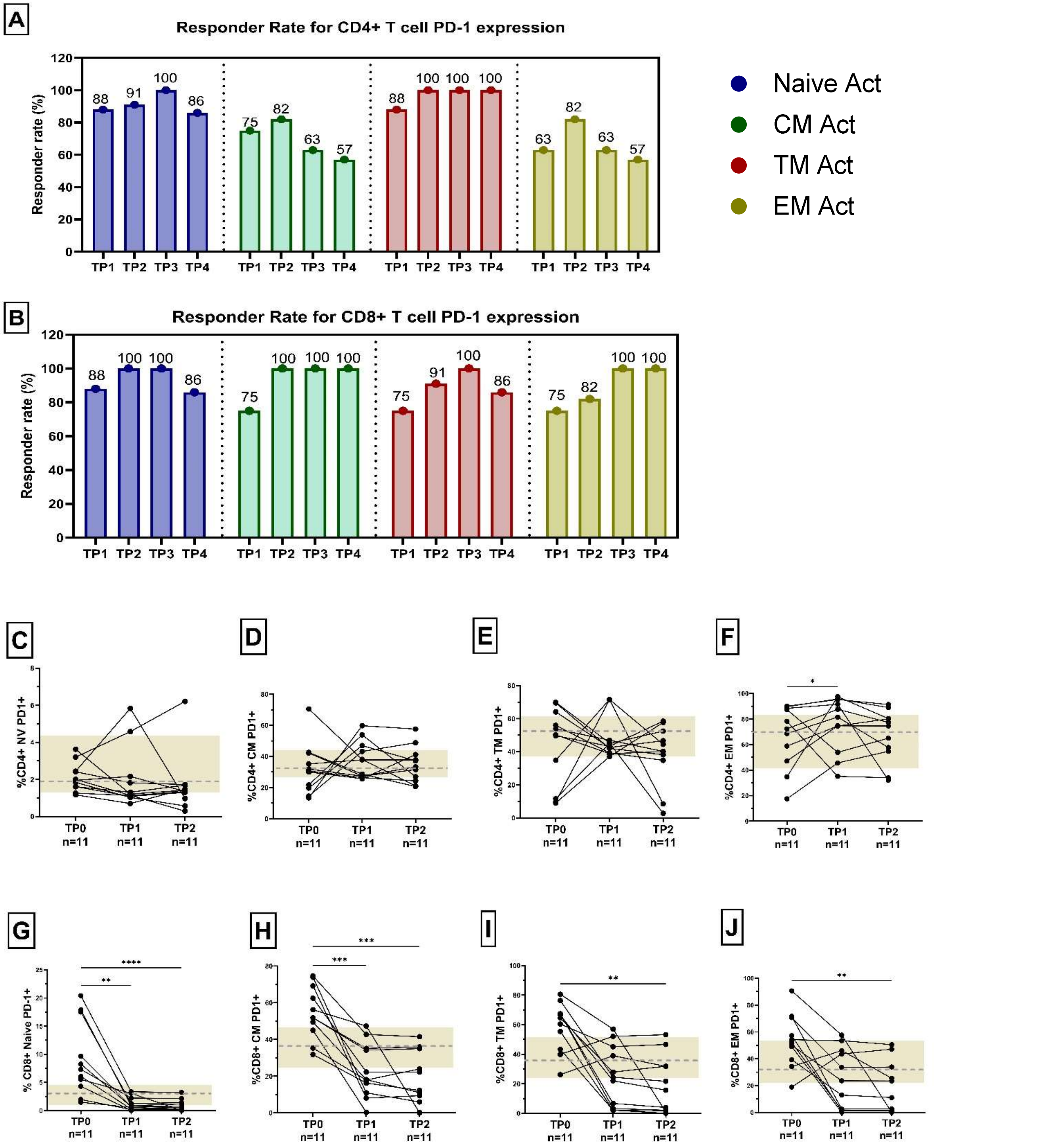

Figure S11: Levels of Tregs and subsets following ART initiation in HLTBI+ individuals: Responder rate for (A) CD4+ Tregs and its subsets. Matched data for frequency of (B) Total Tregs (C) Naïve Tregs and (D) Memory Tregs at TP1, TP2 and TP3. Area shaded in cream color represent frequency of range of sero-negative levels and grey dotted line represent median. Area shaded in cream color represent of range of frequency of HIV sero-negative levels and grey dotted line represent median. Comparison between groups was calculated by Friedman one-way ANOVA non-parametric test, (*p < 0.05; **p < 0.01; ***p < 0.001; ****p < 0.0001)

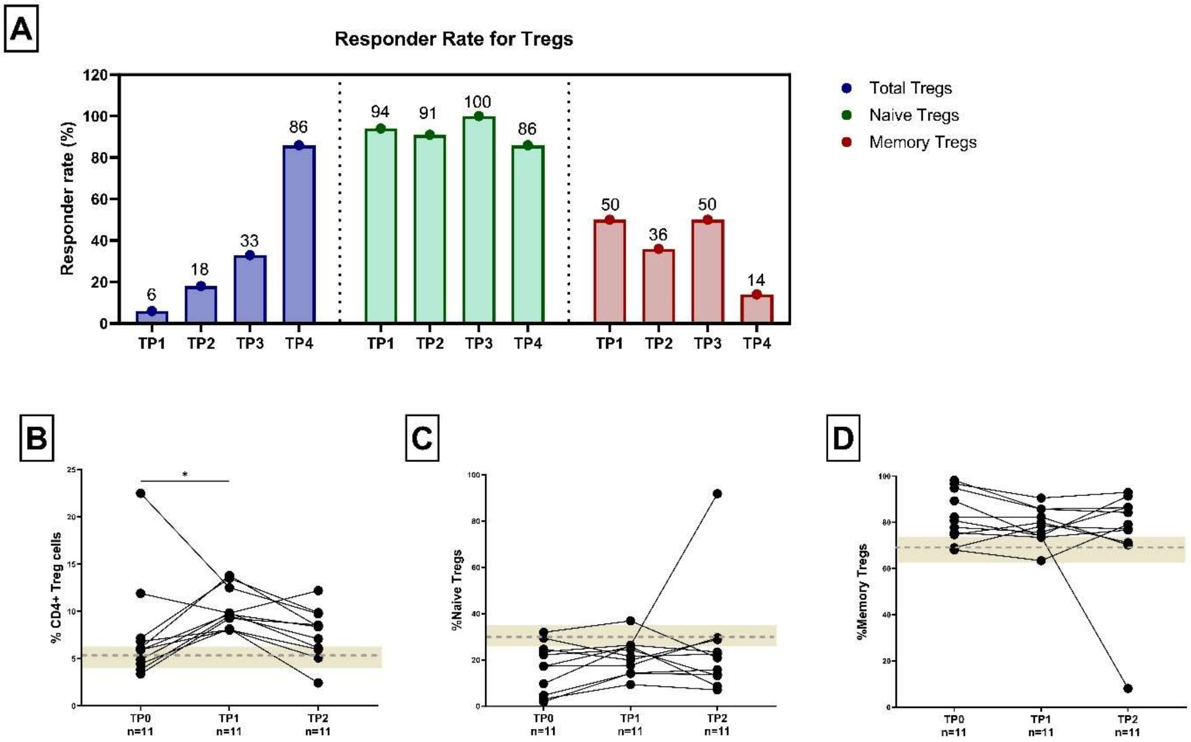

Figure S12: sCD163, CRP, D-dimer and cytokine levels in blood plasma following initiation of ART in HLTBI+ group: Levels of plasma (A) sCD163 (B) CRP in ng/mL and levels of (C) D-dimer (D) IL-10 (E) IL-12p70 (F) IL-17A (G) IP-10 in pg/mL and (H) IFN-γ in pg/mL at TP0 and TP1 in HLTBI+ group. Comparison between matched data for TP0 and TP1 was calculated by Wilcoxon matched-pairs signed rank test (*p < 0.05, **p < 0.01, ***p < 0.001)

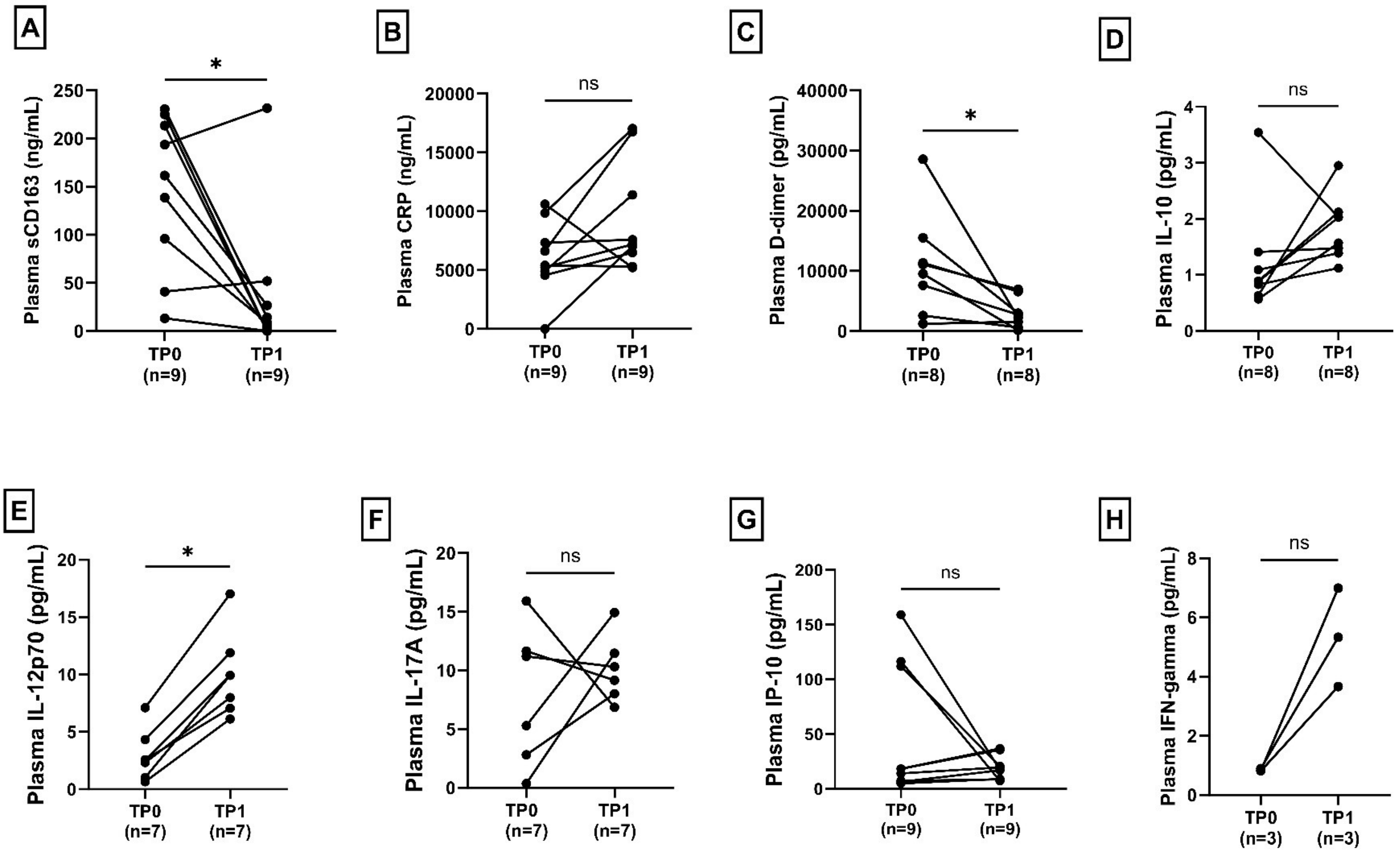
A
*
Plasma sCD163 (ng/mL)
250
200
150
100
50
0
TP0
(n=9)
TP1
(n=9)
B
ns
Plasma CRP (ng/mL)
20000
15000
10000
5000
0
TP0
(n=9)
TP1
(n=9)
C
*
Plasma D-dimer (pg/mL)
40000
30000
20000
10000
0
TP0
(n=8)
TP1
(n=8)
D
ns
Plasma IL-10 (pg/mL)
4
3
2
1
0
TP0
(n=8)
TP1
(n=8)
E
*
Plasma IL-12p70 (pg/mL)
20
15
10
5
0
TP0
(n=7)
TP1
(n=7)
F
ns
Plasma IL-17A (pg/mL)
20
15
10
5
0
TP0
(n=7)
TP1
(n=7)
G
ns
Plasma IP-10 (pg/mL)
200
150
100
50
0
TP0
(n=9)
TP1
(n=9)
H
ns
Plasma IFN-gamma (pg/mL)
8
6
4
2
0
TP0
(n=3)
TP1
(n=3)

Figure S13: Gating strategy for intracellular cytokine staining (ICCS) assay

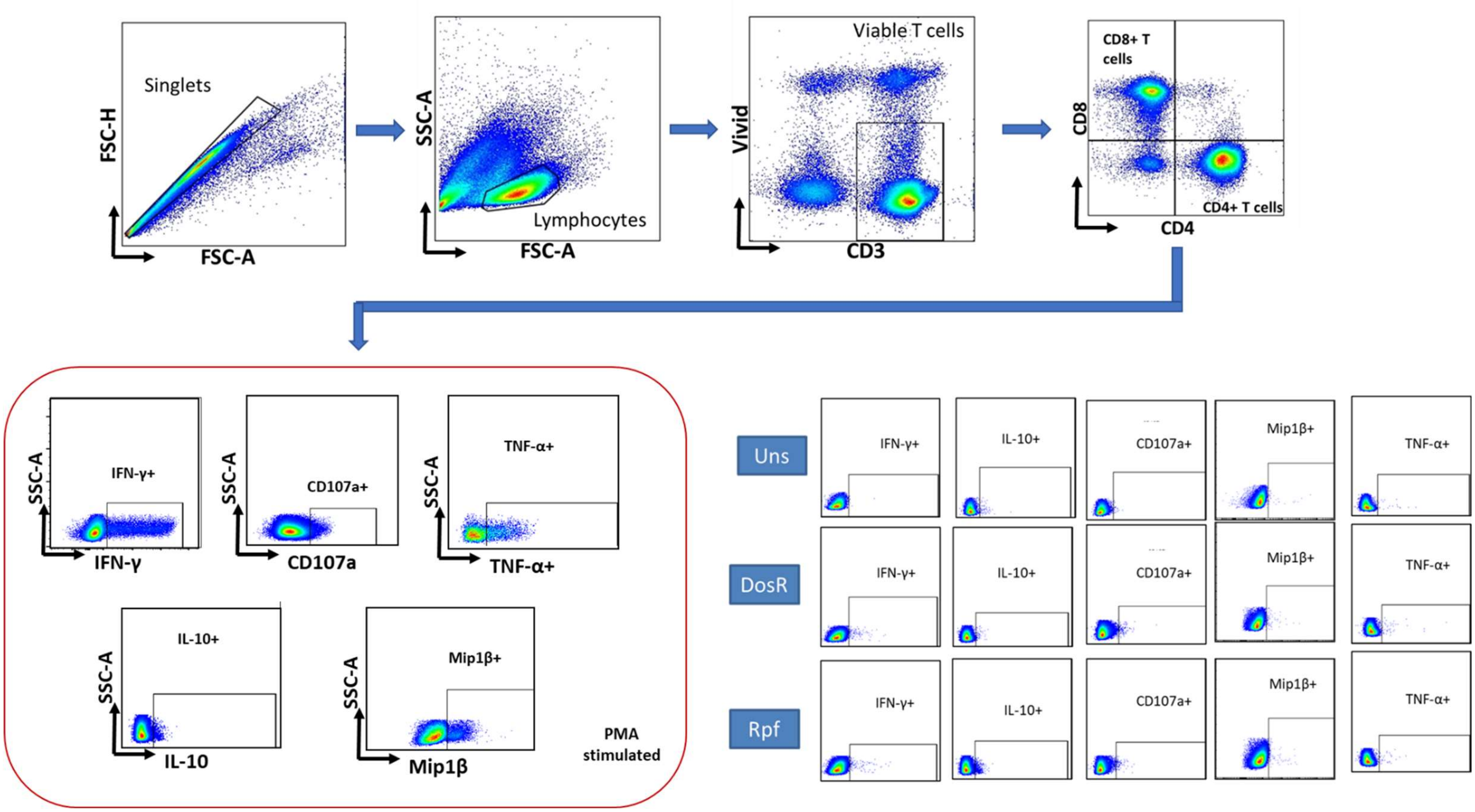

Representative Flow cytometric gating strategy for intracellular cytokine staining (ICCS) assay: First, singlets are gated on the basis FSC-A and FSC-H. Further, lymphocytes are gated on the basis of FSC-A and SSC-A. Following lymphocytes, viable T cells are gated on the basis of expression of CD3 and exclusion of ViViD dye and subsequently CD4+ and CD8+ T cells. For both CD4+ and CD8+ T cells we further gated for 5 functional markers IFN-γ, CD107a, TNF-α, IL-10 and Mip-1β. Uns= Unstimulated, DosR = Stimulation with DosR antigen, Rpf= Stimulation with Rpf antigen

Figure S14: Levels of functional markers against TB specific antigens: Frequency of functional markers for (A) DosR specific CD4+ T cells (B) DosR specific CD8+ T cells (C) Rpf specific CD4+ T cells and (D) Rpf specific CD8+ T cells. Comparison between groups for unmatched data was calculated by Kruskal-Wallis one-way ANOVA non-parametric test, (*p < 0.05, **p < 0.01, ***p < 0.001).

A

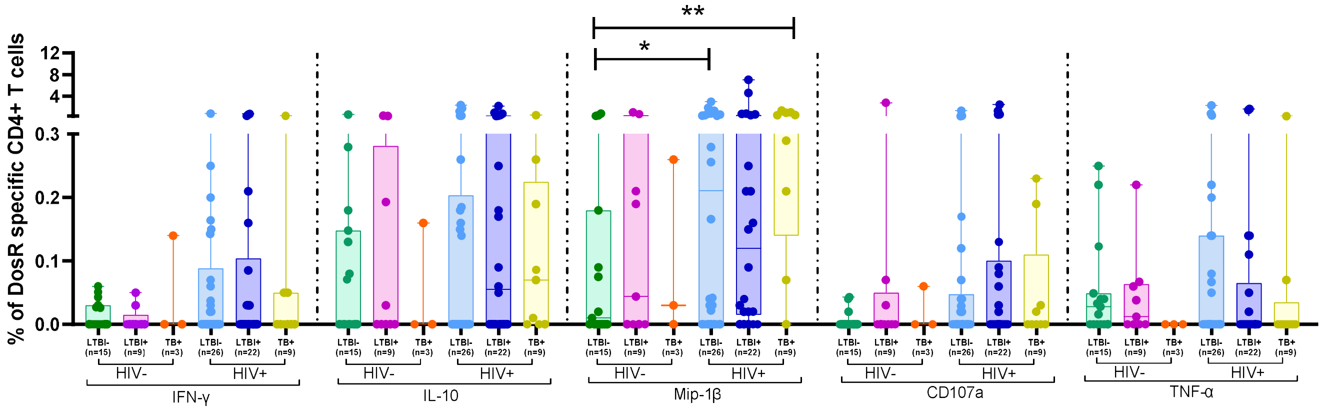

B

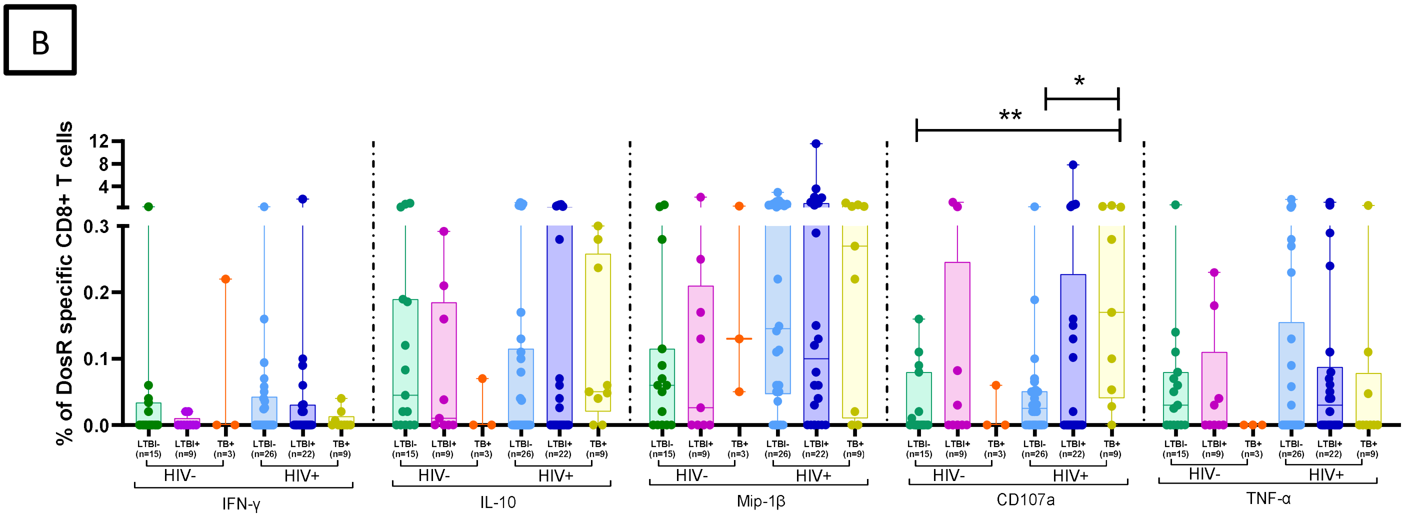

C

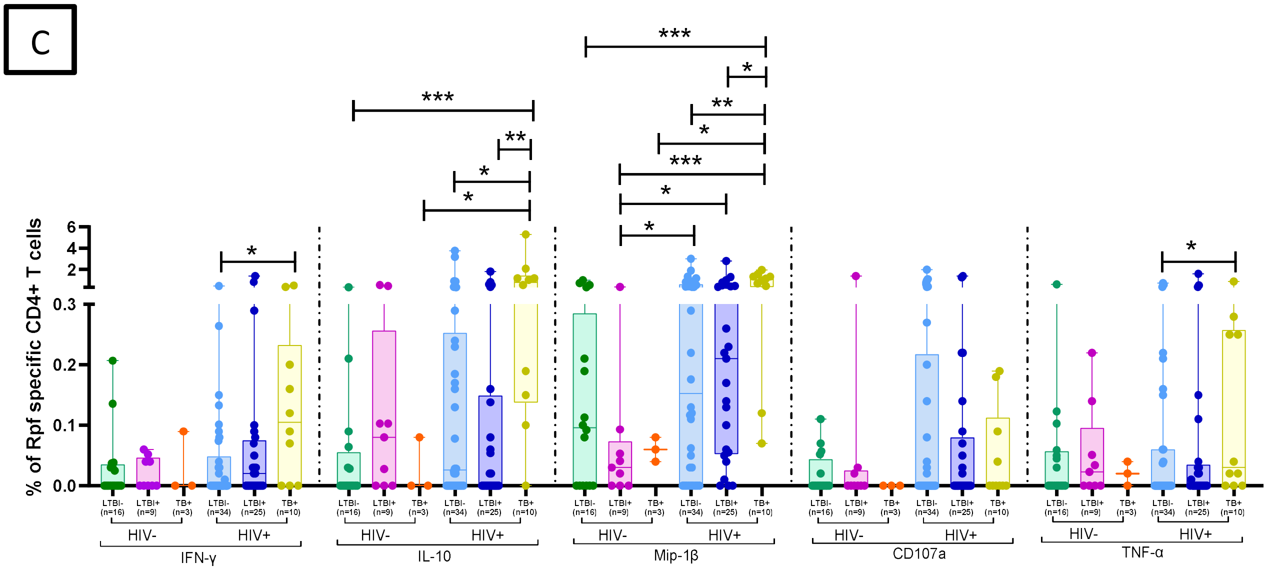
% of Rpf specific CD4+ T cells
LTBI- (n=16)
LTBI+ (n=9)
TB+ (n=3)
LTBI- (n=34)
LTBI+ (n=25)
TB+ (n=10)
HIV-
HIV+
IFN-γ
IL-10
Mip-1β
CD107a
TNF-α

D

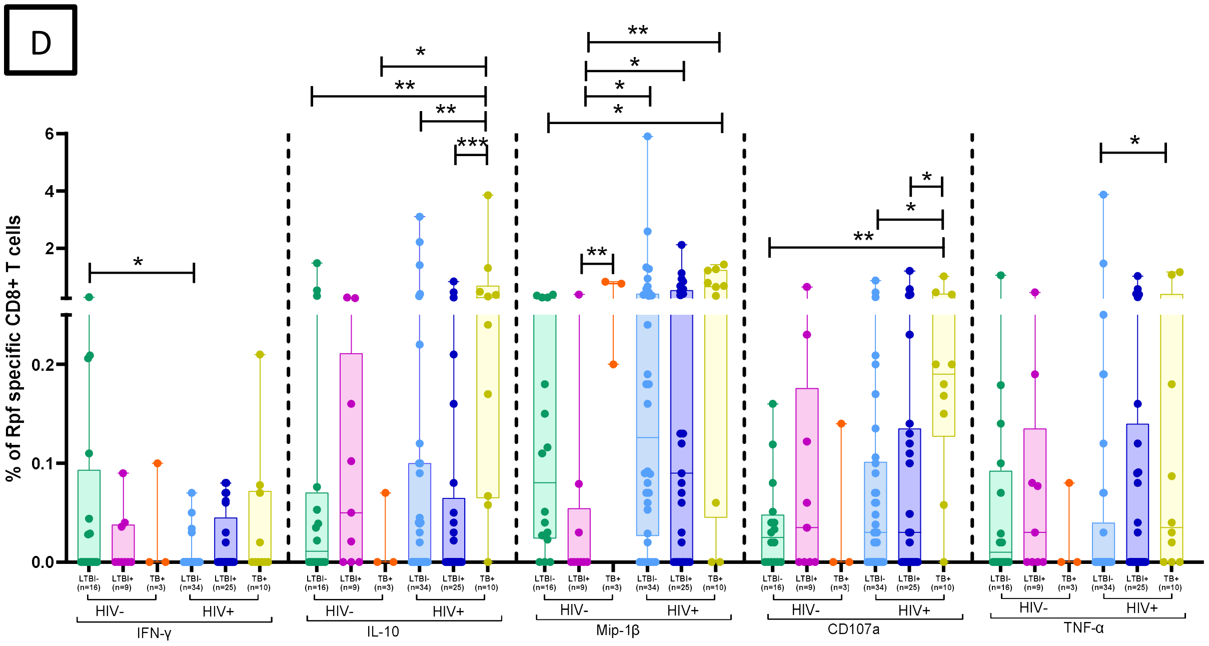
% of Rpf specific CD8+ T cells
LTBI- (n=16)
LTBI+ (n=9)
TB+ (n=3)
LTBI- (n=34)
LTBI+ (n=25)
TB+ (n=10)
HIV-
HIV+
IFN-γ
IL-10
Mip-1β
CD107a
TNF-α

Figure S15: Unmatched data for frequency of functional markers against DosR antigens following ART initiation: Unmatched data for frequency of functional marker response against DosR antigen for (A) IFN-γ (B) IL-10 (C) Mip-1β (D) CD107a and (E) TNF-α within CD4+ T cells. Area shaded in cream color represent range of frequency of sero-negative levels and grey dotted line represent median. Comparison between groups for unmatched data was calculated by Kruskal-Wallis one-way ANOVA non-parametric test, (*p < 0.05, **p < 0.01, ***p < 0.001).

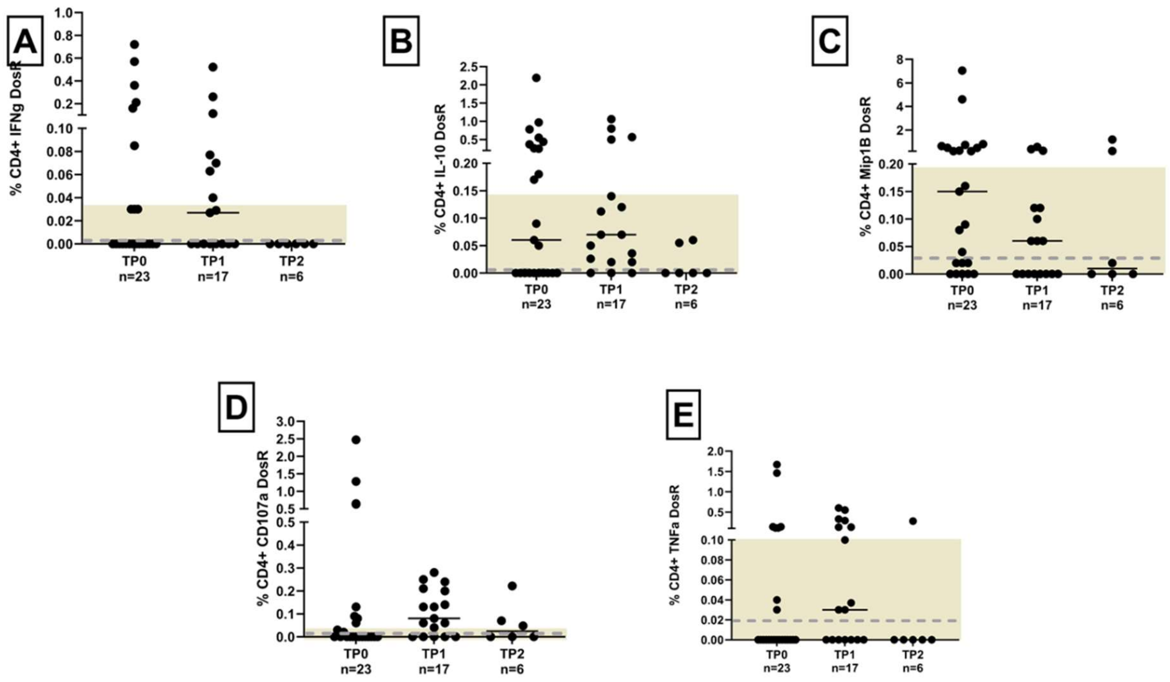

Figure S16: Unmatched data for frequency of functional markers against DosR antigens following ART initiation: Unmatched data for frequency of functional marker response against DosR antigen for (A) IFN-γ (B) IL-10 (C) Mip-1β (D) CD107a and (E) TNF-α within CD8+ T cells. Area shaded in cream color represent range of frequency of sero-negative levels and grey dotted line represent median. Comparison between groups for unmatched data was calculated by Kruskal-Wallis one-way ANOVA non-parametric test, (*p < 0.05, **p < 0.01, ***p < 0.001).

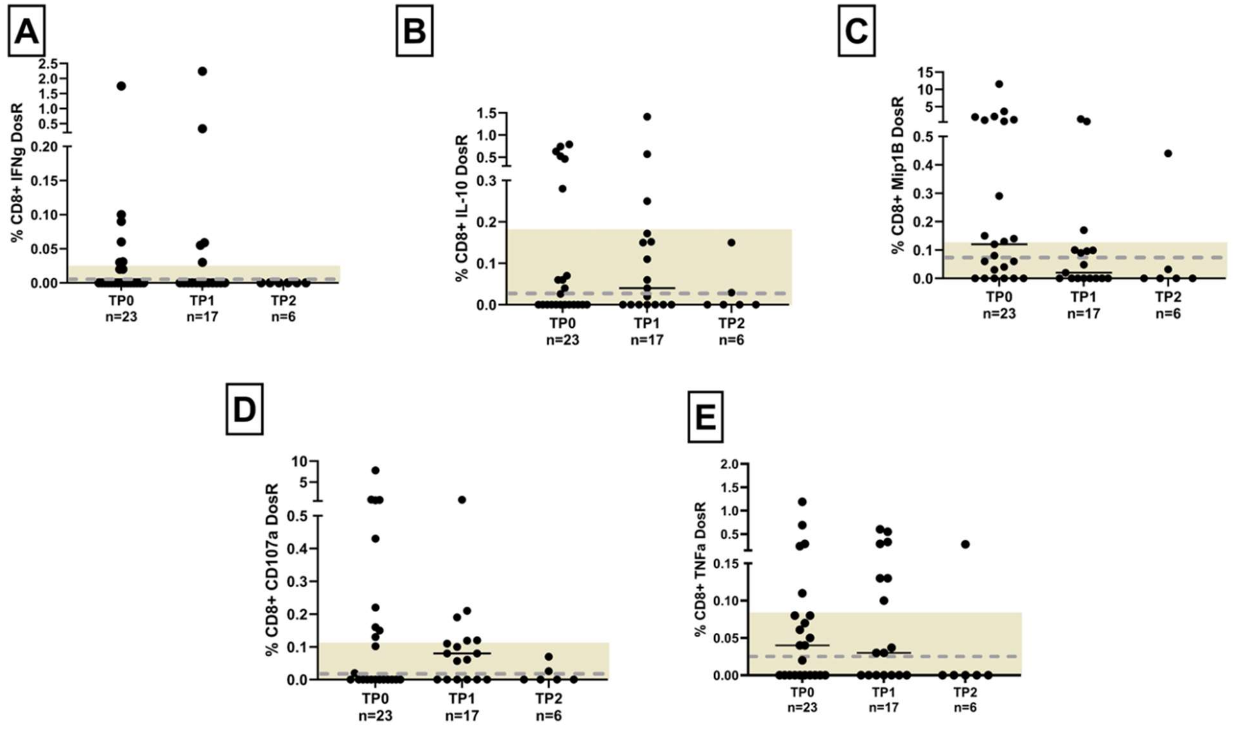

Figure S17: Matched data for frequency of functional markers against DosR antigens following ART initiation: Matched data for frequency of functional marker response against DosR antigen for (A) IFN-γ (B) IL-10 (C) Mip-1β (D) CD107a and (E) TNF-α within CD4+ T cells. Area shaded in cream color represent range of frequency of sero-negative levels and grey dotted line represent median. Comparison between groups for matched data for TP0 and TP1 was calculated by Wilcoxon matched-pairs signed rank test (*p < 0.05, **p < 0.01, ***p < 0.001).

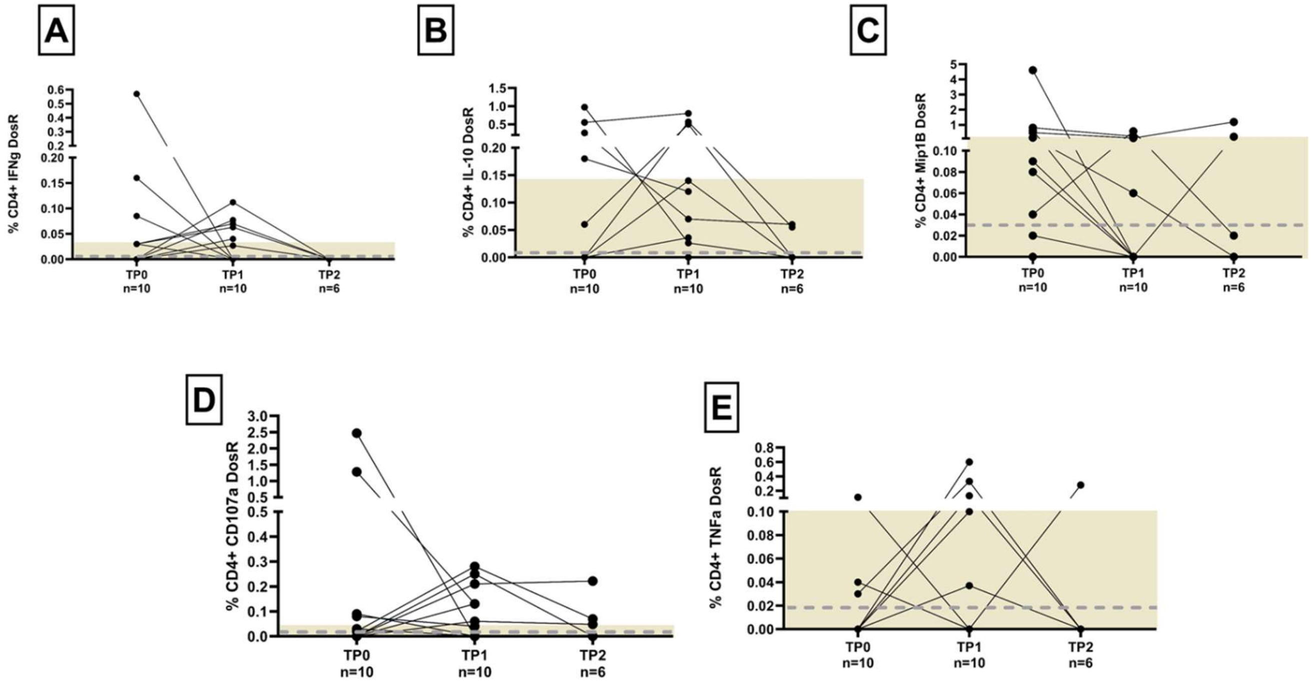

Figure S18: Matched data for frequency of functional markers against DosR antigens following ART initiation: Matched data for frequency of functional marker response against DosR antigen for (A) IFN-γ (B) IL-10 (C) Mip-1β (D) CD107a and (E) TNF-α within CD8+ T cells. Area shaded in cream color represent range of frequency of sero-negative levels and grey dotted line represent median. Comparison between groups for matched data for TP0 and TP1 was calculated by Wilcoxon matched-pairs signed rank test (*p < 0.05, **p < 0.01, ***p < 0.001).

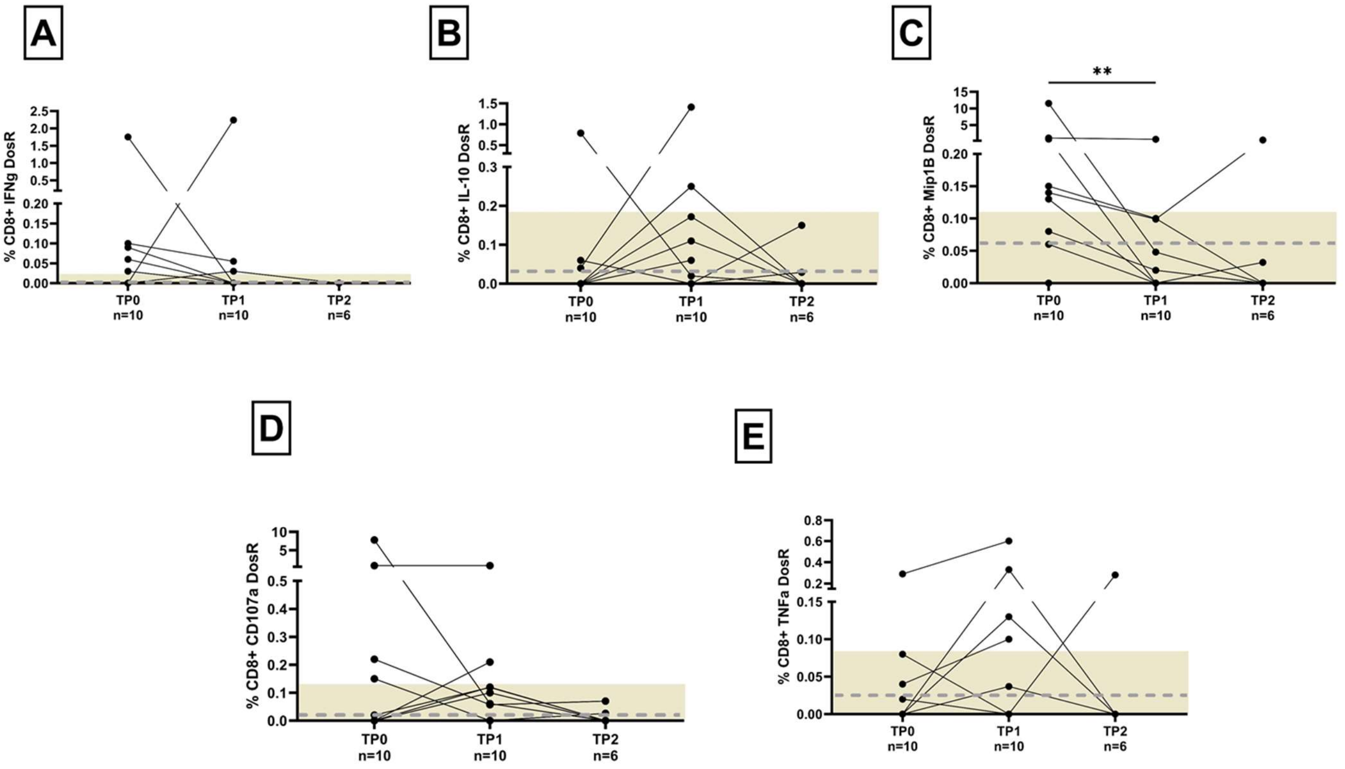

Figure S19: Unmatched data for frequency of functional markers against Rpf antigens following ART initiation: Unmatched data for frequency of functional marker response against Rpf antigen for (A) IFN-γ (B) IL-10 (C) Mip-1β (D) CD107a and (E) TNF-α within CD4+ T cells. Area shaded in cream color represent range of frequency of sero-negative levels and grey dotted line represent median. Comparison between groups for unmatched data was calculated by Kruskal-Wallis one-way ANOVA non-parametric test, (*p < 0.05, **p < 0.01, ***p < 0.001).

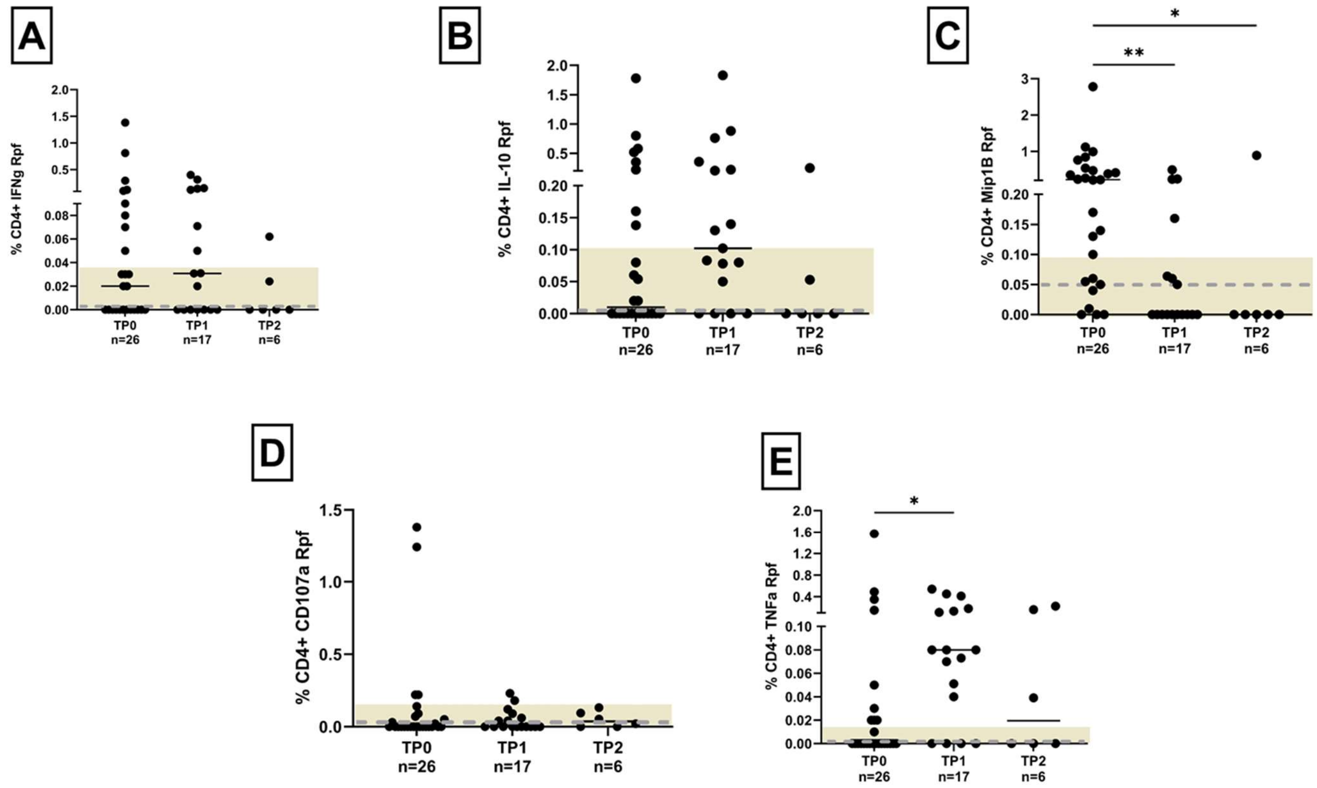

Figure S20: Unmatched data for frequency of functional markers against Rpf antigens following ART initiation: Unmatched data for frequency of functional marker response against Rpf antigen for (A) IFN-γ (B) IL-10 (C) Mip-1β (D) CD107a and (E) TNF-α within CD8+ T cells. Area shaded in cream color represent range of frequency of sero-negative levels and grey dotted line represent median. Comparison between groups for unmatched data was calculated by Kruskal-Wallis one-way ANOVA non-parametric test, (*p < 0.05, **p < 0.01, ***p < 0.001).

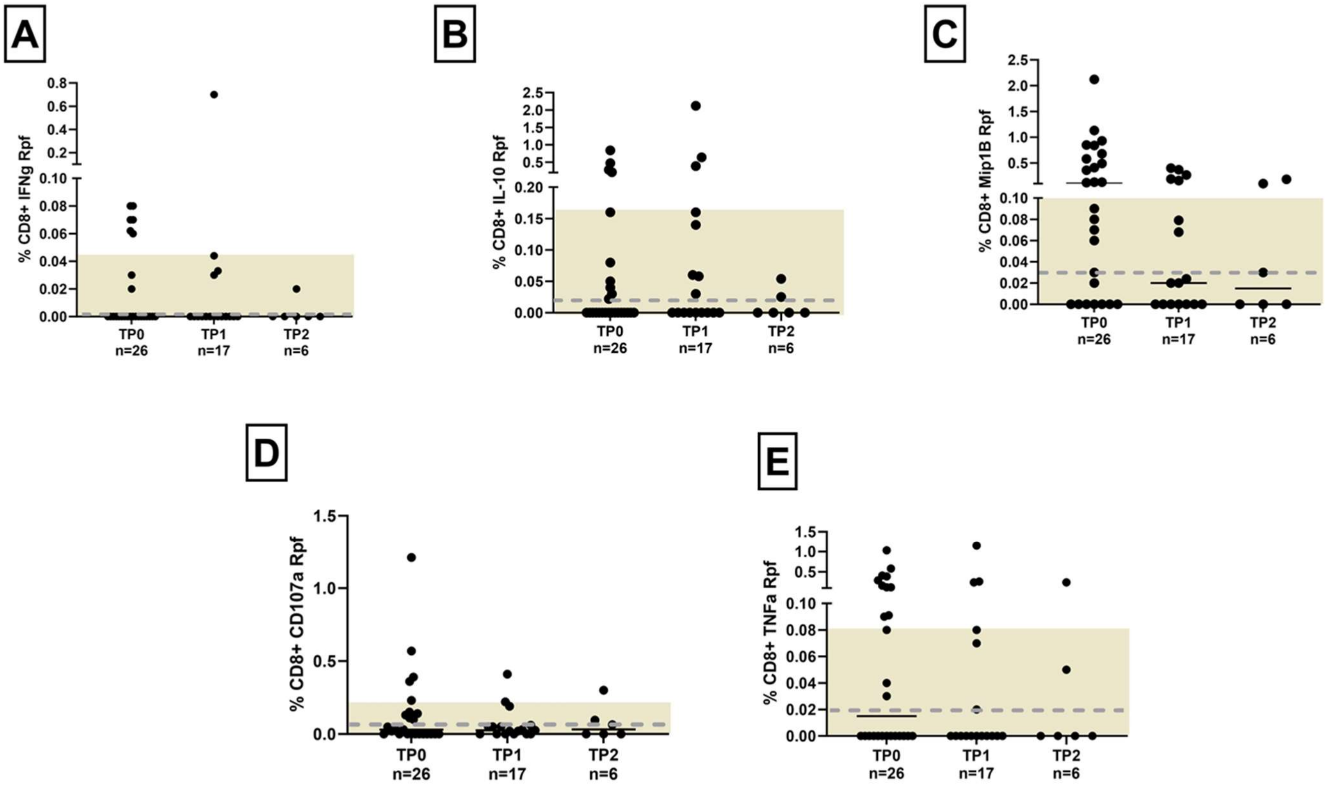

Figure S21: Matched data for frequency of functional markers against Rpf antigens following ART initiation: Matched data for frequency of functional marker response against Rpf antigen for (A) IFN-γ (B) IL-10 (C) Mip-1β (D) CD107a and (E) TNF-α within CD4+ T cells. Area shaded in cream color represent range of frequency of sero-negative levels and grey dotted line represent median. Comparison between groups for matched data for TP0 and TP1 was calculated by Wilcoxon matched-pairs signed rank test (*p < 0.05, **p < 0.01, ***p < 0.001).

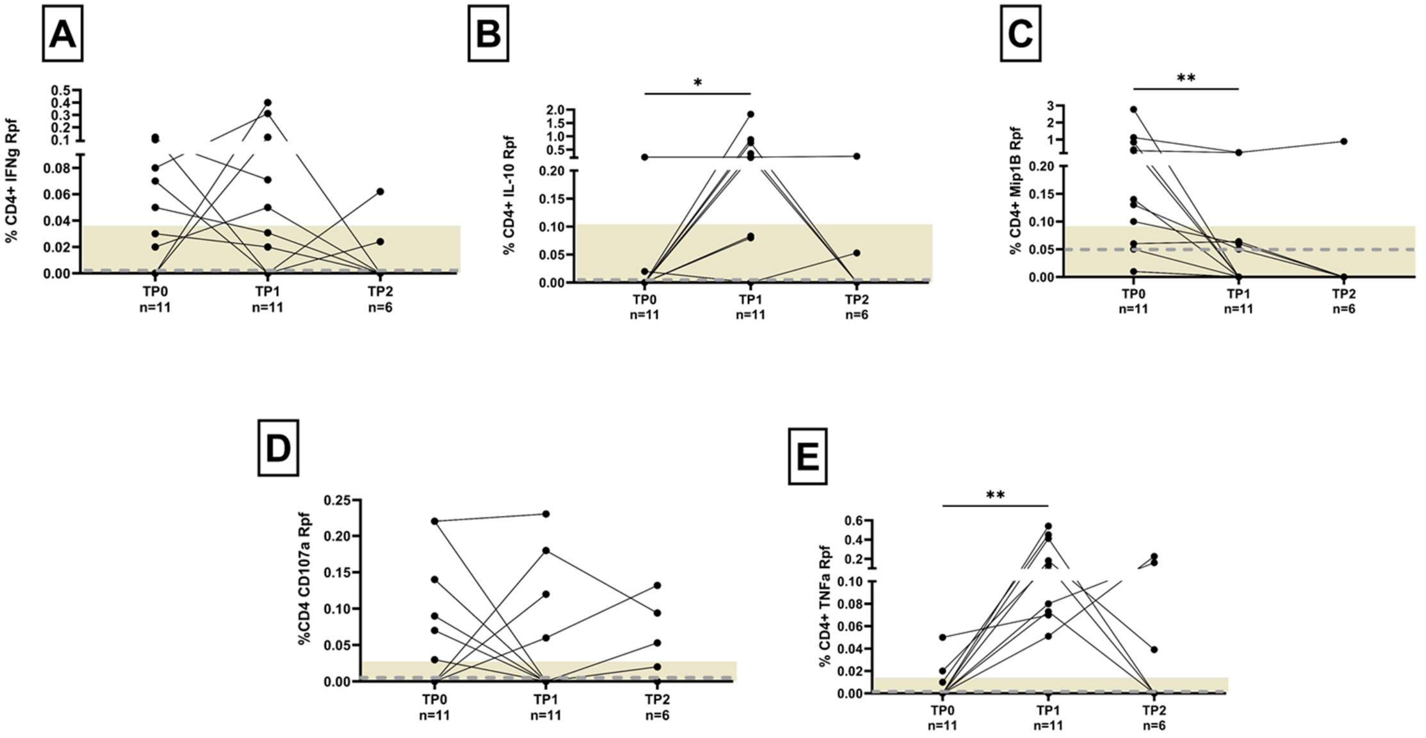

Figure S22: Matched data for frequency of functional markers against Rpf antigens following ART initiation: Matched data for frequency of functional marker response against

Rpf antigen for (A) IFN-γ (B) IL-10 (C) Mip-1β (D) CD107a and (E) TNF-α within CD8+ T cells. Area shaded in cream color represent range of frequency of sero-negative levels and grey dotted line represent median. Comparison between groups for matched data for TP0 and TP1 was calculated by Wilcoxon matched-pairs signed rank test (*$p < 0.05$, **$p < 0.01$, ***$p < 0.001$).

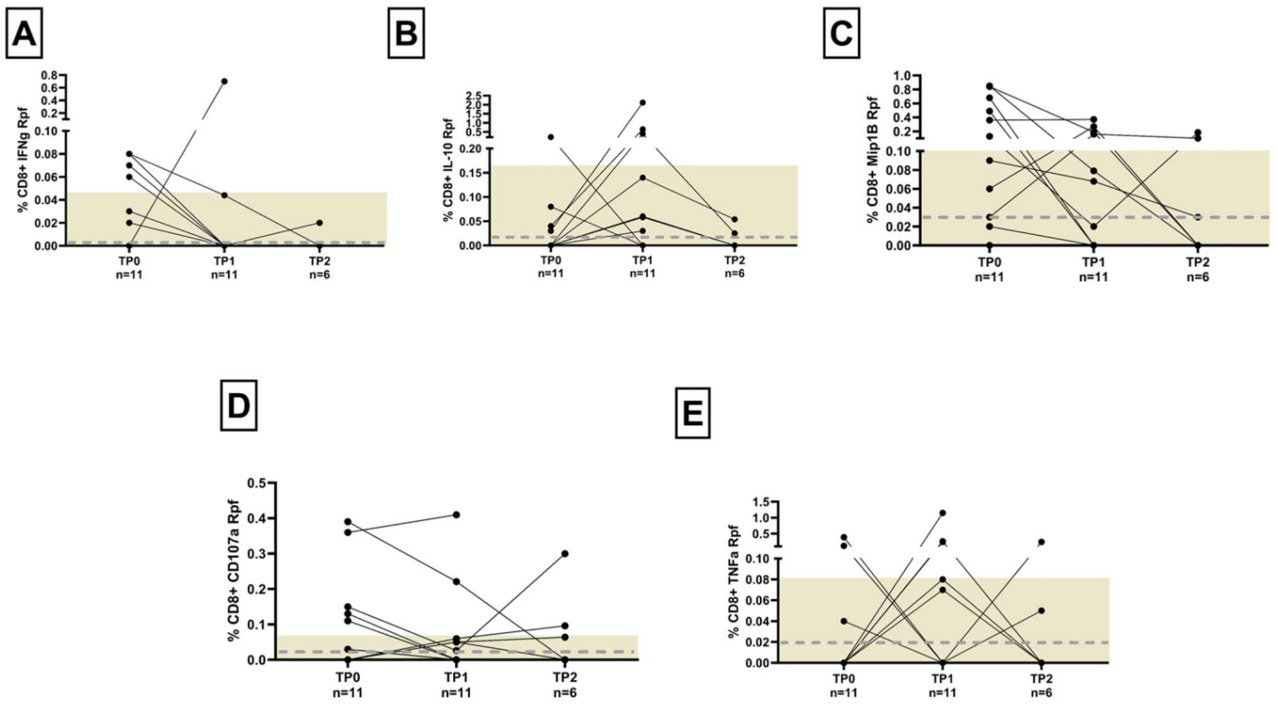

Supplement: Supplementary file 1 [file cells-14-01622-s001.zip › cells-3797682-supplementary.pdf]
